# Rapid photosynthetic induction after short dark periods mitigates growth reduction in lettuce exposed to frequent light-dark cycles

**DOI:** 10.3389/fpls.2026.1791561

**Published:** 2026-03-30

**Authors:** Yuyao Kong, Shuyang Zhen

**Affiliations:** 1 Department of Horticultural Sciences, Texas A&M University, College Station, TX, United States; 2 Department of Horticultural Sciences, University of Florida, Gainesville, FL, United States

**Keywords:** fluctuating light, indoor farming, induction kinetics, leafy greens, LED lighting, photosynthetic efficiency

## Abstract

In indoor crop production, light intensity is typically maintained constant throughout the photoperiod. Dynamic lighting strategies, such as dimming or turning off lights during periods of high electricity prices, can help reduce energy costs. However, fluctuating light conditions may negatively affect plant growth. In this study, we quantified the effects of frequent light-dark cycles on lettuce growth and physiological responses. Two lettuce cultivars, ‘Rex’ and ‘Rouxai’, were grown under seven treatments that differed in the number of light-dark cycles per 24-h period (1–96 cycles): 16 h light/8 h dark (1 cycle; control), 8 h/4 h (2 cycles), 4 h/2 h (4 cycles), 1 h/30 min (16 cycles), 30 min/15 min (32 cycles), 20 min/10 min (48 cycles), and 10 min/5 min (96 cycles). Across all treatments, light intensity during the light periods was maintained at 300 µmol m^-2^ s^-1^. The ‘16 h/8 h’ and ‘8 h/4 h’ treatments resulted in the greatest plant growth in both cultivars. In contrast, the ‘1 h/30 min’ and ‘20 min/10 min’ treatments resulted in the lowest leaf area and shoot biomass in ‘Rouxai’. Plants under these treatments exhibited a slow increase in photosynthetic rate (P_net_) following transitions from dark to light. This slow photosynthetic induction, combined with the frequent light-dark cycles, likely attributed to the reduced growth. Similarly, the ‘20 min/10 min’ treatment resulted in the lowest steady-state P_net_ and shoot mass in ‘Rex’. However, both cultivars grown under the most frequent light-dark cycle (‘10 min/5 min’) showed rapid photosynthetic induction and comparable growth to those grown under the control treatment. Pigment levels, including total chlorophylls, carotenoids, and anthocyanins, decreased under frequent light-dark cycles (32–96 cycles per 24-h). Overall, frequent light-dark cycles reduced lettuce growth and pigmentation. However, when the dark period was brief (5 min), the negative effects on plant growth were mitigated, likely due to rapid photosynthetic induction. The duration of dark periods and cycle frequency should be carefully considered to minimize growth reductions when implementing non-static lighting strategies in indoor production.

## 1 Introduction

In natural environments, plants are continually exposed to fluctuations in light conditions caused by diurnal changes in solar angle, cloud movement, wind, and canopy shading. During transitions from low light or darkness to high light, photosynthesis increases gradually before reaching a steady-state rate, a process termed photosynthetic induction (Chazdon and Pearcy, 1986). Due to biochemical and stomatal limitations, adjustments in photosynthetic activity typically lag behind changes in light intensity, resulting in reductions in light use efficiency and carbon assimilation rate of approximately 10–40% during the induction period compared with steady-state conditions (Pearcy, 1990; Kaiser et al., 2016; Long et al., 2022).

To cope with fluctuating light, plants have evolved various mechanisms that provide photoprotection while modulating photosynthetic efficiency. During sudden increases in light intensity, absorbed energy can temporarily exceed the capacity of photosynthetic carbon assimilation, leading to excess excitation pressure and over-reduction of the photosynthetic electron transport chain (Roach and Krieger-Liszkay, 2014). To protect photosynthetic apparatus from photodamage, excess energy is safely dissipated as heat through non-photochemical quenching (NPQ) (Ware et al., 2015). When light intensity subsequently decreases, thermal dissipation is slowly relaxed, allowing photosynthetic efficiency to increase (Kono and Terashima, 2014). Under fluctuating light, a higher fraction of the absorbed light energy is dissipated via photoprotective mechanisms, often leading to reduced photosynthetic efficiency and plant growth. The balance between maximizing photosynthetic efficiency and maintaining adequate photoprotection is important for overall plant productivity (Kono and Terashima, 2014; Long et al., 2022). Consistent with this, previous studies have shown that rapid relaxation of photoprotective mechanisms increases photosynthesis and crop productivity (Kromdijk et al., 2016).

Beyond short-term regulation, long-term exposure to fluctuating light induces extensive acclimation responses at both molecular and structural levels. Transcriptomic studies have revealed reprogramming of genes related to photosynthesis, photoprotection, primary and secondary metabolisms, and circadian regulation, with responses differing by time of day and leaf development stage (Schneider et al., 2019). At the same time, plants often develop structural and morphological adjustments under fluctuating light, such as reduced leaf thickness and chloroplast anatomy (Vialet-Chabrand et al., 2017; Kaiser et al., 2018; Wei et al., 2021).

The rate of photosynthetic induction is a key determinant of time-integrated carbon assimilation under fluctuating light (Taylor and Long, 2017). Photosynthetic induction is strongly influenced by the duration, frequency, and amplitude of light fluctuations. During transitions from darkness or low light to high light, photosynthetic induction rate is typically dependent on the relaxation of three limiting processes: (1) a rapid phase in which limitations on ribulose 1, 5-bisphosphate (RuBP) regeneration are relaxed as the photosynthetic electron transport rate increases within seconds to 1–2 minutes; (2) activation of Calvin cycle enzymes, particularly Rubisco, which is generally completed within ~10 minutes; and (3) the slow opening of stomata that typically require 10–60 minutes to reach full stomatal conductance (Pearcy et al., 1996). Importantly, the relaxation kinetics of these processes depend on the prior light history of the leaf, for example, how long leaves have remained in darkness. Short dark periods allow partial retention of Rubisco activation and stomatal opening, enabling faster photosynthetic induction upon dark-to-light transition (Woodrow and Mott, 1989). However, longer dark periods lead to greater Rubisco deactivation and stomatal closure, thereby prolonging induction time (Woodrow and Mott, 1989).

Plant responses to fluctuating light can vary widely among species and across different light regimes. In *Arabidopsis thaliana*, acclimation to fluctuating light mimicking natural conditions reduced leaf thickness, growth rate, and photosynthetic capacity (Vialet-Chabrand et al., 2017). In tomato, exposure to brief lightflecks (peak light intensity of 1000 μmol m^-2^ s^-1^ for 20 s) every 5 min over 16 h per day for three weeks accelerated photosynthetic induction after darkness compared with plants grown under constant light (Kaiser et al., 2016). More recently, Zhang et al. (2020) examined tomato responses to sinusoidal fluctuating light and observed thinner leaves and lower NPQ, although stomatal conductance, photosynthetic capacity, and overall plant growth were not significantly affected. These studies have primarily focused on sunflecks or stepwise changes in light intensity occurring over seconds to tens of minutes. In contrast, few studies have examined long-term exposure to repeated square-wave light regimes with frequent light-dark cycles, which are relevant for developing cost-effective lighting strategies in indoor crop production.

In indoor farming, constant light levels are usually implemented throughout the photoperiod using electric lighting, such as light emitting diodes (LEDs). Despite the high crop productivity and various environmental benefits, the energy required for crop lighting and environmental control remains a major cost of indoor farming, limiting the economic viability and wider adoption (Kozai, 2022). Implementing dynamic lighting, such as dimming or turning off lights during periods of high electricity prices, can help reduce energy costs (Bhuiyan and van Iersel, 2021). However, studies examining the effects of frequent light-dark cycles on photosynthetic physiology and plant growth under controlled environments are limited. Therefore, the objectives of this study were to: (1) quantify the effects of fluctuating light with different frequencies of light-dark cycles on lettuce growth and morphology, and (2) examine the associated photosynthetic responses and the mechanisms linking photosynthetic responses to plant growth.

## 2 Materials and methods

### 2.1 Plant materials and growing conditions

Two lettuce (*Lactuca sativa*) cultivars, green butterhead ‘Rex’ and red oakleaf ‘Rouxai’, were used in this study. Seeds (Johnny’s Selected Seeds, Fairfield, ME, USA) were sown in 0.9 L plastic pots (10 cm × 10 cm × 9 cm) filled with a soilless substrate containing sphagnum peat moss (80-90%) and perlite (PRO-MIX LP15, Premier Tech Horticulture, Rivière-du-Loup, Canada). The containers were moved into an air-conditioned walk-in growth room after sowing and placed under seven light treatments (see details below). Six containers per cultivar were included in each treatment. Seedlings were thinned to one plant per container and fertigated daily with a nutrient solution made with deionized water, a water-soluble fertilizer (12N-1.75P-13.3K; Jack’s Nutrients FeED 12-4–16 RO; JP Peters, Inc., Allentown, PA, USA), and Epsom salt (MgSO_4_). The nutrient solution contained (in mg L^-1^) 150 N, 21.9 P, 166.2 K, 87.6 Ca, 36.9 Mg, 15 S, 0.255 B, 0.255 Cu, 1.875 Fe, 0.6 Mn, 0.015 Mo, and 0.45 Zn. The pH of the nutrient solution was adjusted to 5.8 using a 0.5 M potassium hydroxide solution.

The air temperature and relative humidity in the growth room were measured with a temperature and humidity probe (EE08-SS, Apogee Instruments, Inc., Logan, UT), and the CO_2_ level was measured with a CO_2_ probe (GMP252, Vaisala Inc., Louisville, CO) throughout the experiment. Additionally, type-J thermocouples were installed to monitor air temperature in each treatment area. All sensors were connected to a data logger (CR1000X, Campbell Scientific, Inc., Logan, UT). Data was measured every 30 seconds and recorded as hourly and daily averages. A small air mixing fan (model CFM-9238B-140-473; Mouser Electronics, Mansfield, TX) was installed in each experimental unit to improve air circulation throughout the experiment. Air temperature was uniform across treatments with minimal variation. The average air temperature was 26.1 ± 0.59 °C (mean ± SD), relative humidity was 51.7 ± 4.5%, and CO_2_ level was 482.4 ± 72.7 μmol mol^-1^.

### 2.2 Light treatments

**Figure 1:**
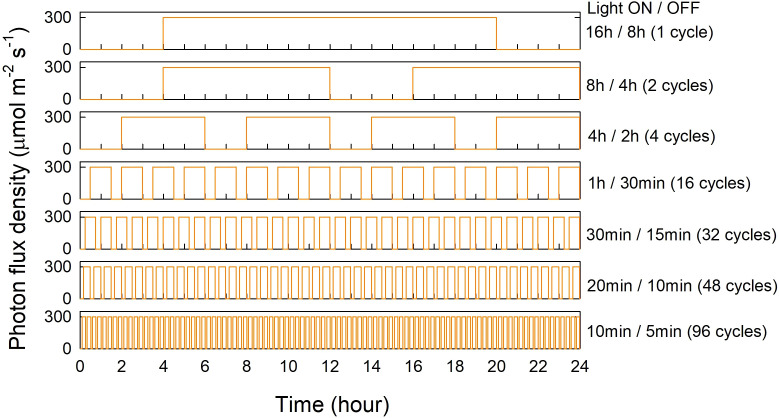
Schematic depiction of seven different light treatments ranging from 1 to 96 light-dark cycles per 24 hours. White light emitting diodes (LEDs) were used to implement the following treatments: 16 h light/8 h dark (one cycle per 24-h), 8 h light/4 h dark (two cycles per 24-h), 4 h light/2 h dark (four cycles per 24-h), 1 h light/30 min dark (16 cycles per 24-h), 30 min light/15 min dark (32 cycles per 24-h), 20 min light/10 min dark (48 cycles per 24-h), and 10 min light/5 min dark (96 cycles per 24-h). The ratio of light to dark duration within each cycle was 2:1 in all treatments. The average photosynthetic photon flux density (PPFD) was 300 µmol m^-2^ s^-1^ in all treatments during the light periods, and the daily light integral (DLI) in each treatment was 17.28 mol m^-2^ d^-1^.

**Figure 2:**
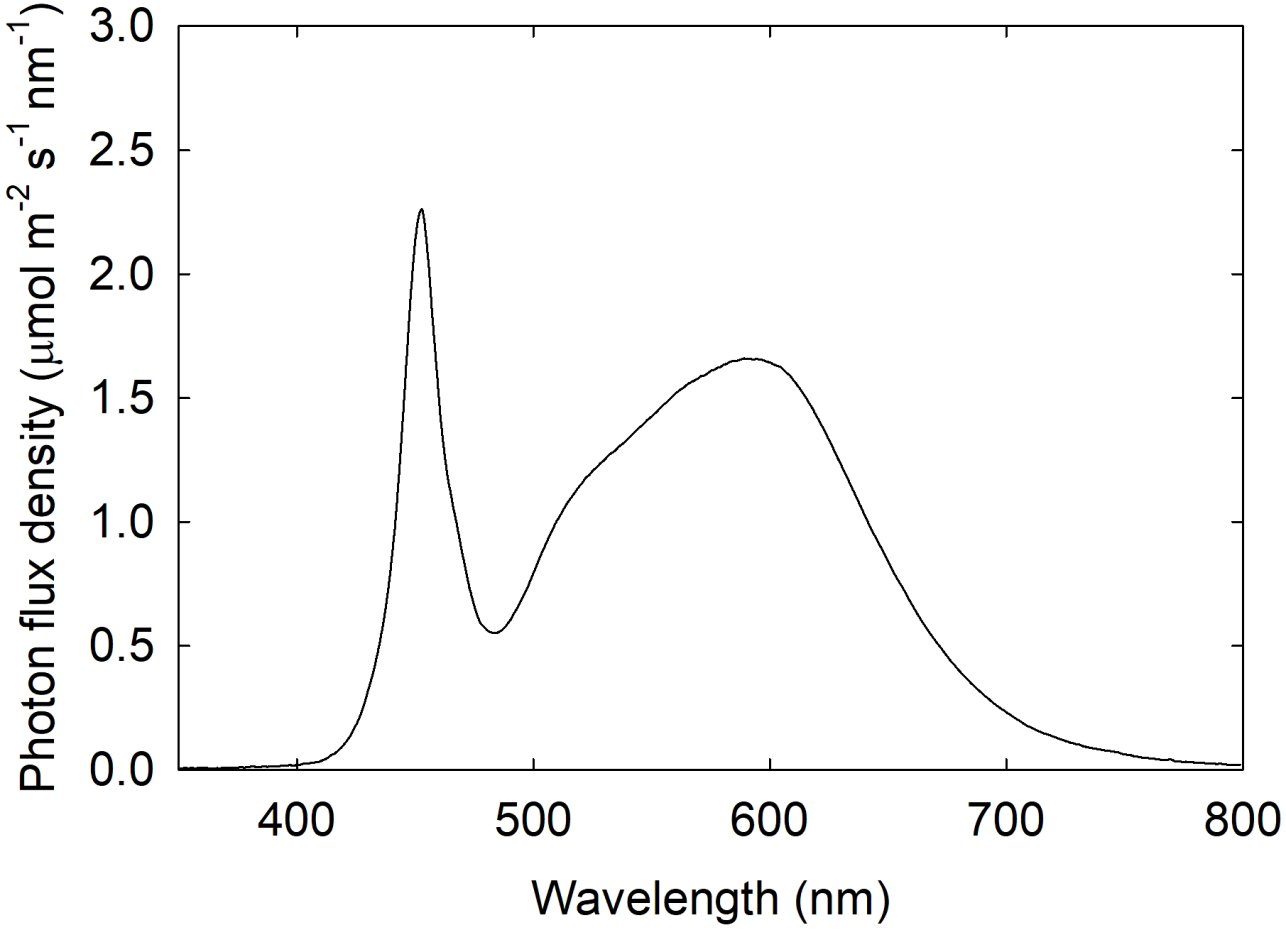
Light spectral distribution of white light emitting diode (LED) light (peak wavelengths at 453 nm and 591 nm).

Lettuce plants were exposed to seven fluctuating light treatments with varying frequencies of light-dark cycles (ranging from 1 to 96 cycles) per 24 hours (**Figure 1**). The light treatments included: 16 h light and 8 h dark (one cycle per 24-h, ‘16 h/8 h’; control), 8 h light and 4 h dark (two cycles per 24-h, ‘8 h/4 h’), 4 h light and 2 h dark (four cycles per 24-h, ‘4 h/2 h’), 1 h light and 30 min dark (16 cycles per 24-h, ‘1 h/30 min’), 30 min light and 15 min dark (32 cycles per 24-h, ‘30 min/15 min’), 20 min light and 10 min dark (48 cycles per 24-h, ‘20 min/10 min’), and 10 min light and 5 min dark (96 cycles per 24-h, ‘10 min/5 min’). In all treatments, the ratio of light to dark duration within each cycle was kept at 2:1, resulting in a cumulative light duration of 16 h and cumulative dark period of 8 h per day in all treatments. The light treatments were randomly assigned to seven experimental compartments (83 cm × 61 cm × 61 cm), which were created using two double-layer metal shelves. The shelves were lined with opaque plastic sheeting, and the compartments were separated using reflective foils to minimize light contamination among the treatments. Each light treatment was provided by two light emitting diode (LED) fixtures (Alina Luminaires, RAYN Growing Systems, Middleton, WI) installed at the top of each treatment compartment. The light fixtures were programmed to emit broad-spectrum white light (peak wavelengths at 453 nm and 591 nm; **Figure 2**), following the light on/off cycles described above. The blue (400–500 nm):green (501–600 nm):red (601–700 nm):far-red (701–800 nm) ratio of the white light was 24.1:44.7:28.7:2.5 (each component expressed as % of total photons within the 400–800 nm range). Photosynthetic photon flux density (PPFD; 400–700 nm) was measured at canopy level (40 cm below the light fixtures) using a spectroradiometer (PS300; Apogee Instruments, Logan, UT) at 12 locations in each light treatment area. The average PPFD was maintained at 300 µmol m^-2^ s^-1^ in all treatments during the light periods. The daily light integral (DLI) in each treatment was 17.28 mol m^-2^ d^-1^. Plants were stacked using layers of foam boards, which were periodically removed to maintain a consistent distance between the top of the plant canopies and the light fixtures.

Plants within each treatment were rotated every 1–2 days to minimize potential effects of spatial variation in light intensity (the standard deviation of the light intensity in each treatment ranged from 48.8 to 60.6 µmol m^-2^ s^-1^).

### 2.3 Leaf photosynthetic responses to light-dark cycles

Leaf photosynthetic responses to light-dark cycles were measured at 27–32 days after planting (DAP). The measurements were made on the most recently fully expanded leaves using a portable gas exchange system with a 2 × 3 cm clear-top chamber (LI-6800; LI-COR, Lincoln, NE). Plants were measured under white light provided by the same type of LEDs described above (**Figure 2**). Light intensity at the leaf surface inside the clear-top chamber was measured using a spectroradiometer (PS300; Apogee Instruments) and adjusted to 300 µmol m^-2^ s^-1^.

During measurements, the LEDs were programmed to match the same light-dark cycle as each corresponding light treatment. Photosynthetic responses were continuously monitored over a light-dark-light cycle for five light treatments with relatively short cycles: ‘4 h/2 h’, ‘1 h/30 min’, ‘30 min/15 min’, ‘20 min/10 min’ and ‘10 min/5 min’. For example, in the ‘1 h/30 min’ treatment, photosynthetic responses were measured during 1 hour of light, followed by 30 min of dark, and then one additional hour of light, to determine the steady-state net photosynthetic rate (P_net_) and how quickly photosynthesis reached steady state during the induction period (i.e., the dark to light transition). In the ‘4 h/2 h’ treatment, photosynthetic responses were measured only during the last hour of the 4-h light period, followed by 2 hours of dark, and then one additional hour of light, due to the longer cycle duration. For the ‘16 h/8 h’ and ‘8 h/4 h’ treatments, leaf P_net_ was measured only during the light phase due to the long duration of each light-dark cycle. Throughout the measurement period, plants being measured followed the same light and dark schedule as those in the corresponding light treatments, ensuring that the photosynthetic responses remained representative and unaffected by the measurement process.

Gas exchange data were recorded every 30 s at the steady-state and every 5 s during the light-to-dark and dark-to-light transition phases to capture the response kinetics. The air temperature within the leaf cuvette was maintained at 25 °C, CO_2_ concentration was 450 µmol mol^-1^, leaf vapor pressure deficit was 1.3 kPa, and flow rate was 700 µmol s^-1^. In each replicate study, measurements were repeated three times on three different plants per lettuce cultivar per treatment. The average leaf P_net_ was calculated from the response curves for three intervals: the first 10 minutes of the light period, the entire light period of a single light-dark cycle, and the steady-state phase under each light treatment.

### 2.4 Leaf light response curves and chlorophyll fluorescence measurements

Leaf light response curves were constructed at 33–35 DAP on three plants per cultivar for only three light treatments (‘16 h/8 h’, ‘1 h/30 min’ and ‘10 min/5 min’) due to the short interval between when the leaves of those lettuce cultivars became sufficiently large and sturdy for clamping in the gas exchange chamber and when plants reached mature, harvestable size. These treatments were selected to represent a range of light-dark cycles frequencies, from least to most frequent. Measurements were conducted on the most recently fully expanded leaves using a portable gas exchange system equipped with a fluorometer (LI-6800; LI-COR). The fluorometer provided a light source containing 80% red light and 20% blue light with intensity ranging from 0 to 1500 µmol m^-2^ s^-1^. Plants were first given 30 min to acclimate to a light intensity of 1500 µmol m^-2^ s^-1^. Net photosynthetic rate was then measured in descending order of light levels: 1500, 1200, 800, 500, 250, 100, 50, 0 µmol m^-2^ s^-1^. At each light level, plants were given 5 to 6 min for photosynthetic rate to reach a steady state. The CO_2_ concentration within the cuvette was maintained at 450 µmol mol^-1^, and leaf vapor pressure deficit was 1.4 kPa.

Chlorophyll fluorescence was measured on most recently fully expanded leaves to detect any stress or photodamage induced by the fluctuating light cycles. Plants were dark-adapted for 2 hours before measurements with a fluorometer (OS1p; Opti-Science, Hudson, NH). The maximum quantum yield of photosystem II (PSII) was estimated as the ratio of variable fluorescence to maximum fluorescence (F_v_/F_m_). Plants were harvested twice in each replicate study (see details below). Chlorophyll fluorescence measurements were made one day before each harvest, at 4 weeks and 5 weeks after planting, on both lettuce cultivars.

### 2.5 Chlorophyll and carotenoid concentrations

Chlorophyll and total carotenoid concentrations were measured for each plant by sampling three leaf disks from light exposed regions of the most recently mature leaves using a cork borer (8.43 mm in diameter). The leaf disks were immediately put into 5 mL dimethyl sulfoxide solution and bathed at 65 °C until the disks became transparent. The extracted solution was then measured at 480 nm, 649.1 nm, and 655.1 nm for light absorbance using a spectrophotometer (GENESYS™ 180 UV–Visible Light; Thermo Fisher Scientific, Waltham, MA). The chlorophyll and total carotenoid concentrations (µg mL^-1^) were calculated following the equations by Wellburn (1994).

Chlorophyll a (Chl_a_) concentration = 12.47 × A_655.1_ − 3.62 × A_649.1_
Chlorophyll b (Chl_b_) concentration = 25.06 × A_649.1_ − 6.5 × A_655.1_
Total carotenoid concentration = (1000 × A_480_ − 1.29 × Chl_a_ − 53.78 × Chl_b_)/220

The concentration of chlorophylls in µg mL^-1^ was converted to micromoles per square meter of leaf area (µmol m^-2^) using the molar mass of chlorophyll a or chlorophyll b, and the leaf disk area per sample. The concentration of total carotenoids was converted to milligrams per square meter of leaf area (mg m^-2^). The chlorophyll and total carotenoid concentrations were measured at both harvests.

### 2.6 Anthocyanin concentration

Anthocyanin concentration was quantified using both extraction and image analysis methods at each harvest. For the extraction method, five representative leaf disks were collected per plant from leaves that were fully exposed to light using a cork borer with 9.41 mm diameter. The leaf disks were immediately transferred into vials containing 5 mL extraction solution (5% 3M HCl + 15% H_2_O + 80% C_2_H_6_O) (Gould et al., 2000) and then placed in a refrigerator at 6 °C for 16 h. Subsequently, light absorbance of the extracted solution was measured at 530 nm and 653 nm using the spectrophotometer (GENESYS™ 180 UV-Visible Light; Thermo Fisher Scientific). The anthocyanin index was determined using the method described by Gould et al. (2000):

Anthocyanin index = A_530 nm_ − A_653 nm_ × 0.24.

For image analysis method, top-down photos were taken before each harvest using a digital camera positioned 152 cm above the plants. The RGB images were analyzed using an open-source Python program to determine the normalized difference anthocyanins index (NDAI), an index developed by Kim and van Iersel (2023) for estimating anthocyanins concentration. NDAI is calculated based on the optical properties of anthocyanins, specifically their high absorptance in the green and low absorptance in the red region of the spectrum. The Python program distinguishes the plant objects from the background and extracts the pixel intensity of the green and red color channels from the RGB images (**Supplementary Figure 1**). NDAI is then calculated as (I_red_ − I_green_)/(I_red_ + I_green_), where I represents the pixel intensity (Kim and van Iersel, 2023). Image-based NDAI analysis was conducted for the red oakleaf cultivar ‘Rouxai’ only.

### 2.7 Growth parameters

To quantify the effects of fluctuating light cycles on plant growth at different stages, lettuce plants were harvested twice, at 4 weeks (young plant stage) and 5 weeks (mature stage) after planting. Three plants per cultivar from each light treatment were harvested each time. At harvest, shoot fresh mass (g plant^-1^) of each plant was measured. Total leaf area (cm^2^ plant^-1^) of each plant was determined using a leaf area meter (LI-3100C; LI-COR, Lincoln, NE). Then, plants were oven-dried at 60 °C for five days to obtain shoot dry mass (g plant^-1^). Leaf mass per area (LMA, g cm^-2^) was calculated as the ratio of shoot dry mass (g plant^-1^) to total leaf area (cm^2^ plant^-1^). The occurrence of tip burn was assessed through visual observations, with severity graded on a scale from 0 to 5, where 0 indicated the absence of tip burn and 5 represented the most severe form of tip burn.

### 2.8 Statistical analysis

The experiment was conducted three times following a randomized complete block design. Each replicate was treated as a block and the light treatments were randomized in each replication. Light response curves were fitted using nonlinear regression (exponential rise to maximum) in SigmaPlot (SigmaPlot 15.0; Systat Software Inc., Palo Alto, CA). Data were analyzed using Analysis of Variance (ANOVA) in Statistical Analysis Systems (SAS Institute, Cary, NC). Mean separation was performed using Fisher’s least significant difference test (LSD, *P* < 0.05). Data from the two lettuce cultivars were analyzed separately.

## 3 Results

### 3.1 Total leaf area and plant biomass

**Figure 3:**
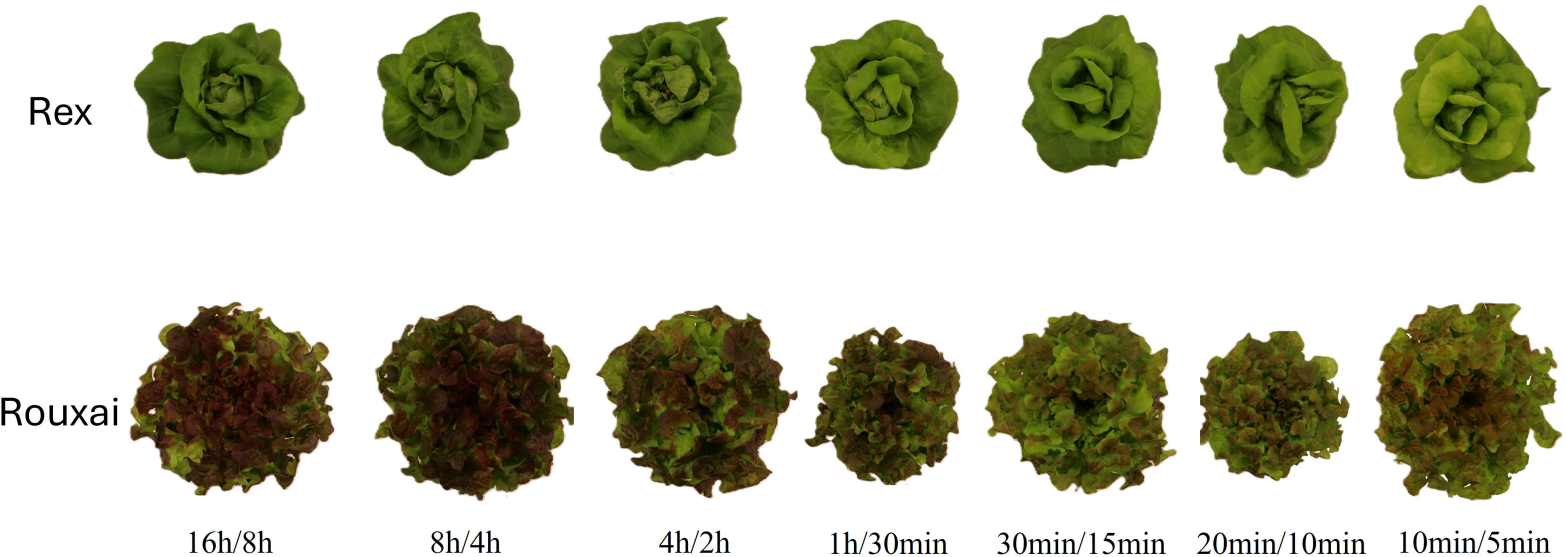
Representative lettuce plants of ‘Rex’ (top) and ‘Rouxai’ (bottom) grown under different light-dark cycle treatments. See **Figure 1** legend for treatment details.

**Figure 4:**
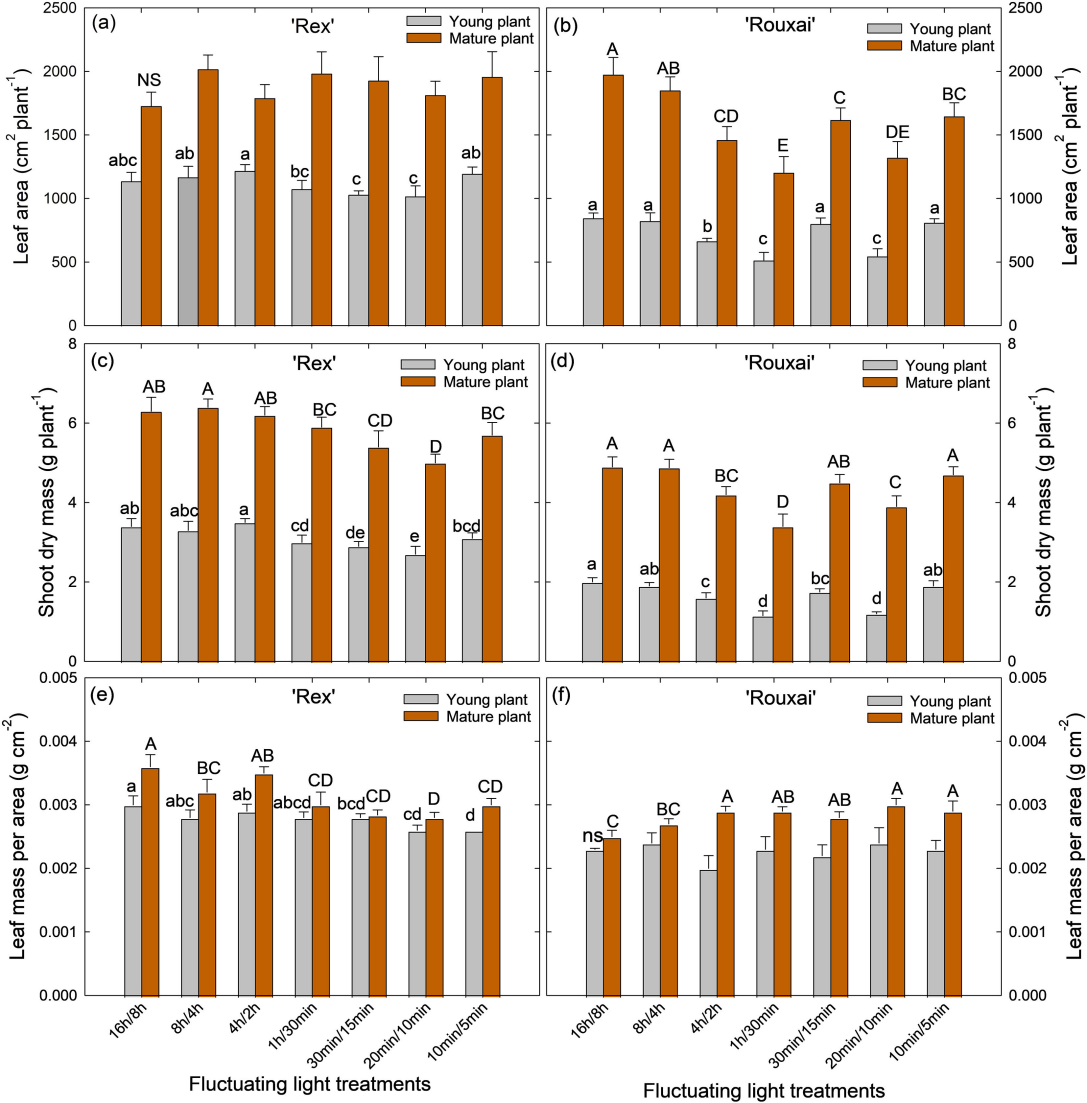
Total leaf area **(a, b)**, shoot dry mass **(c, d)**, and leaf mass per area **(e, f)** of ‘Rex’**(a, c, e)** and ‘Rouxai’ **(b, d, f)** under different light-dark cycles. See **Figure 1** legend for treatment details. Within each plant growth stage, different letters (uppercase for the mature plant stage and lowercase for young plant stage) indicate significance at *P* < 0.05 among the treatments, with error bars representing SE (n = 9; 3 plants per replicate x 3 replicate studies). NS stands for non-significance.

At the young-plant stage (harvested 4 weeks after sowing), light treatments with fewer light-dark cycles per 24-h period generally resulted in greater total leaf area and shoot biomass (**Figures 3**, **4**; **Supplementary Figure 2**). The ‘16 h/8 h’ (control) and ‘8 h/4 h’ treatments, which had only one and two light-dark cycles per 24-h, respectively, resulted in the highest total leaf area and shoot fresh and dry mass in both cultivars (**Figure 4**; **Supplementary Figure 2**). The ‘4 h/2 h’ treatment (four cycles per 24-h) resulted in similar total leaf area and shoot fresh and dry mass in ‘Rex’ young plants as those in the ‘16 h/8 h’ and ‘8 h/4 h’ treatments but significantly reduced plant growth in ‘Rouxai’. Treatments with16 to 48 light-dark cycles per 24-h (‘1 h/30 min’, ‘30 min/15 min’, and ‘20 min/10 min’) tended to reduce leaf area and shoot biomass in both cultivars, with greater reductions under more frequent cycles in ‘Rex’. However, total leaf area of ‘Rouxai’ young plants under the ‘30 min/15min’ treatment did not differ significantly from the control, and plant growth was greater than in the ‘1 h/30 min’ treatment and the ‘20 min/10min’ treatments (**Figures 4b, d**). Interestingly, lettuce grown under the most frequent light-dark cycles (‘10 min/5 min’; 96 cycles per 24-h) showed total leaf area and shoot biomass comparable to those under the ‘16 h/8 h’ and ‘8 h/4 h’ treatments in both cultivars (**Figure 4**; **Supplementary Figure 2**).

At the mature plant stage (harvested at 5 weeks after sowing), there were no significant differences in total leaf area among treatments in ‘Rex’ (**Figure 4a**). However, shoot fresh and dry mass exhibited similar treatment responses to young plants, with highest values under treatments with fewer dark-light cycles per 24-h period (‘16 h/8 h’, ‘8 h/4 h’, and ‘4 h/2 h’) and increasingly greater reductions under more frequent cycles (‘1 h/30 min’ ‘30min/15 min’, and ‘20 min/10 min’) (**Figure 4c**; **Supplementary Figure 2a**). The ‘10 min/5 min’ treatments had similar shoot mass as the control. Both total leaf area and shoot biomass in mature ‘Rouxai’ plants showed similar responses as young plants. The highest total leaf area and shoot fresh and dry mass were observed in the ‘16 h/8 h’ and ‘8 h/4 h’ treatments, while the lowest values were found in the ‘1 h/30 min’ and ‘20 min/10 min’ treatments. Compared to the control, the ‘10 min/5 min’ treatment resulted in significantly reduced leaf area (by 16.6%) but similar shoot fresh and dry mass (**Figures 4b, d**; **Supplementary Figure 2b**).

The effects of different light-dark cycles on LMA differed between ‘Rex’ and ‘Rouxai’. In ‘Rex’, the highest LMA was observed under the ‘16 h/8 h’ (control) treatment in both young and mature plants (**Figure 4e**). Treatments with 16 to 96 cycles per 24-h (‘1 h/30 min’, ‘30 min/15 min’, ‘20 min/10 min’, and ‘10 min/5 min’) resulted in the lowest LMA, although there were no significant differences among these treatments (**Figure 4e**). In contrast, in mature ‘Rouxai’ plants, more frequent cycles (16–96 cycles per 24-h) resulted in the highest LMA, whereas the control treatment resulted in the lowest LMA (**Figure 4f**). LMA did not differ significantly among treatments in young ‘Rouxai’ plants (**Figure 4f**).

### 3.2 Pigment concentrations

**Figure 5:**
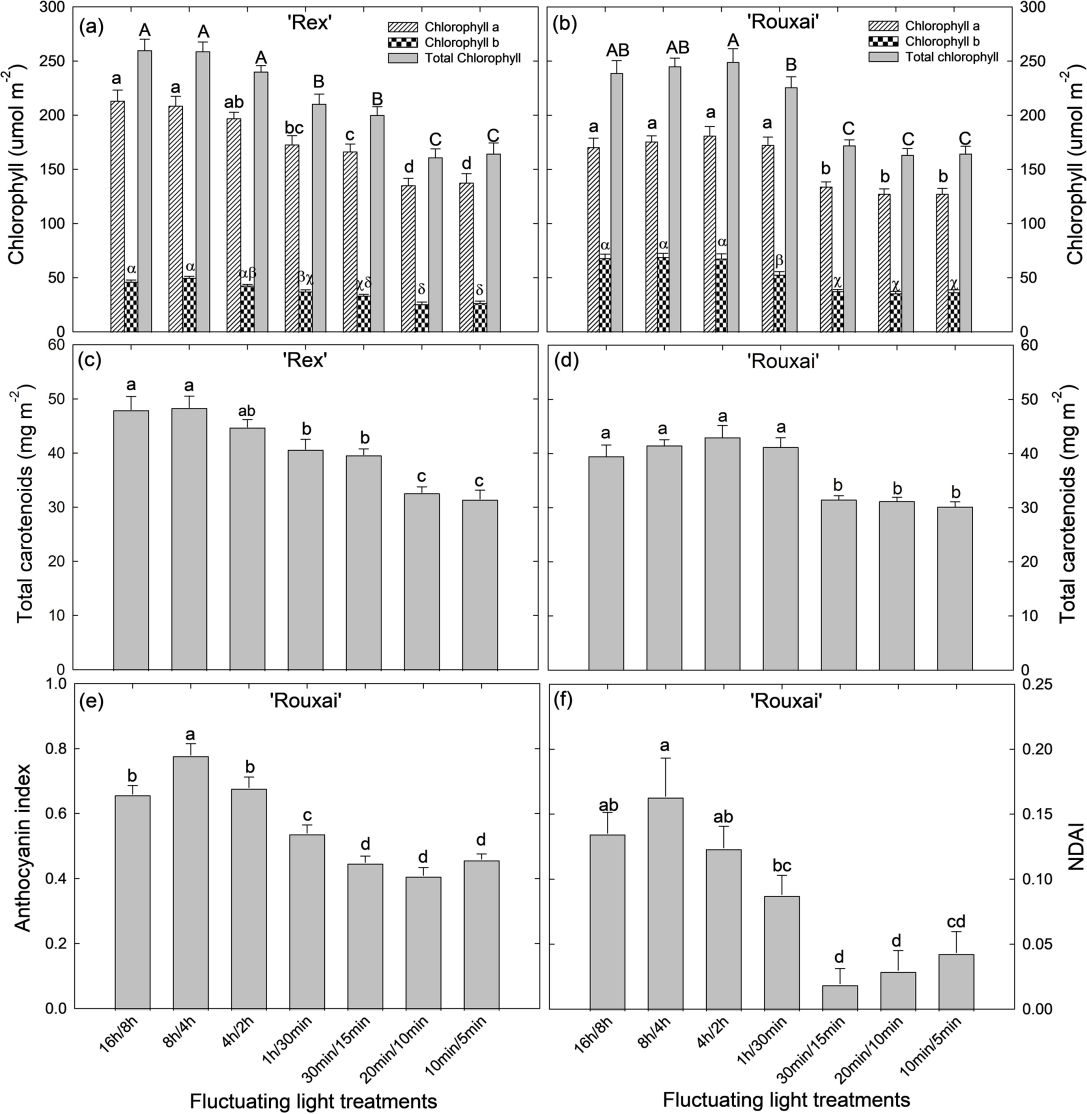
Chlorophylls concentration **(a, b)** and total carotenoids concentration **(c, d)** of ‘Rex’ **(a, c)** and ‘Rouxai’ **(b, d)** at the young plant stage, as well as extraction-based anthocyanin index **(e)** and image-based normalized difference anthocyanin index (NDAI) **(f)** of ‘Rouxai’ young plants under different light-dark cycles. See **Figure 1** legend for treatment details. Different letters indicate significance at *P* < 0.05 among the treatments with error bars representing SE (n = 9; 3 plants per replicate x 3 replicate studies).

Pigment concentrations, including chlorophylls, total carotenoids, and anthocyanins, decreased under more frequent light-dark cycles in both cultivars (**Figures 3**, **5**; **Supplementary Figure 3**). Specifically, in ‘Rex’ young plants, chlorophyll a, chlorophyll b, total chlorophylls, and total carotenoids concentrations were highest under the less frequent light-dark cycles (‘16 h/8 h’, ‘8 h/4 h’, and ‘4 h/2 h’) and reduced progressively under more frequent cycles (**Figures 5a, c**). The most frequent light-dark cycles treatments (‘20 min/10 min’ and ‘10 min/5 min’) resulted in the lowest chlorophylls and total carotenoids concentrations (**Figures 5a, c**). Similarly, in ‘Rouxai’ young plants, chlorophylls and total carotenoids concentrations were significantly reduced in the ‘30 min/15 min’, ‘20 min/10 min’ and ‘10 min/5 min’ treatments compared to treatments with less frequent light/dark cycles (**Figures 5b, d**).

Anthocyanins concentration was the highest in the ‘8 h/4 h’ treatment in ‘Rouxai’ young plants, followed by the ‘16 h/8 h’ control treatment, and lowest in the ‘30 min/15 min’, ‘20 min/10 min’ and ‘10 min/5 min’ treatments, based on both the anthocyanins index obtained from anthocyanins extraction and the NDAI values from image analysis (**Figures 5e, f**). A close correlation was observed between anthocyanin concentrations estimated from extraction and image-based NDAI values (analysis not shown), indicating that the extraction method reliably reflected anthocyanin responses at the whole-canopy level.

Similar trends in chlorophyll a, chlorophyll b, total chlorophylls, total carotenoids, and anthocyanins concentrations were observed in mature plants of both cultivars (**Supplementary Figure 3**).

### 3.3 Leaf photosynthetic responses

#### 3.3.1 Leaf photosynthetic responses to light-dark cycles

**Figure 6:**
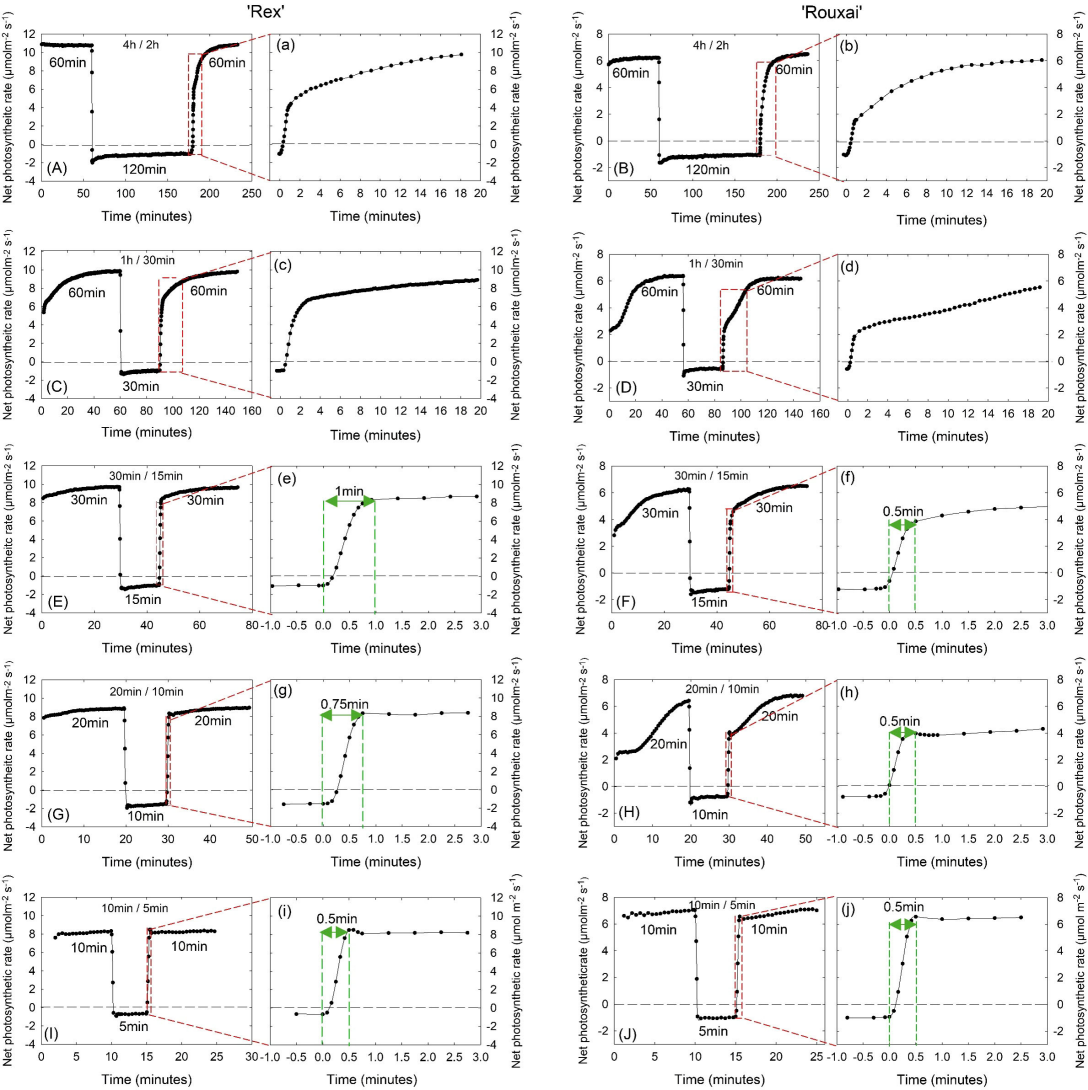
Representative leaf photosynthetic responses of ‘Rex’ **(A, C, E, G, I)** and ‘Rouxai’ **(B, D, F, H, J)** to light-dark cycles under the 4 h/2 h, 1 h/30 min, 30 min/15 min, 20 min/10 min, and 10 min/5 min treatments. Light intensity was maintained at 300 µmol m^-2^ s^-1^ during light periods. See **Figure 1** legend for treatment details. Panels **(a)**, **(c)**, **(e)**, **(g)**, and **(i)**, as well as **(b)**, **(d)**, **(f)**, **(h)**, and **(j)**, show expanded views of the photosynthetic responses during the light induction phase corresponding to each main panel. The x-axis was rescaled such that time 0 indicates the initiation of the light period following the preceding dark period in each treatment. Note that the x-axis timescales differ among treatments.

*Induction kinetics.* Different light/dark cycles markedly affected leaf photosynthetic responses in both lettuce cultivars. Both ‘Rex’ and ‘Rouxai’ plants grown in the ‘4 h/2 h’ and ‘1 h/30 min’ treatments exhibited slow induction kinetics during the dark-to-light transition (**Figures 6A–D**); P_net_ gradually increased for more than 20 minutes before reaching a relatively steady state (**Figures 6a–d**). In contrast, photosynthetic rate of ‘Rex’ grown in the ‘30 min/15 min’, ‘20 min/10 min’, and ‘10 min/5 min’ treatments showed rapid increases during the light induction phase (**Figures 6E, G**). Time to reach a near-steady-state maximum P_net_ was approximately 1.0 min, 0.75 min, and 0.5 min in the ‘30 min/15 min’, ‘20 min/10 min’, and ‘10 min/5 min’ treatments, respectively (**Figures 6e, g**). Similarly, P_net_ in ‘Rouxai’ plants grown in the ‘30 min/15 min’ and ‘20 min/10 min’ treatments increased rapidly over ~0.5 min upon transition from dark to light (**Figures 6f, h**). However, P_net_ continued to increase slowly over an additional ~20 minutes to before reaching a maximum (**Figures 6F, H**). In the ‘10 min/5 min’ treatment, ‘Rouxai’ plants showed a rapid induction similar to ‘Rex’, with P_net_ quickly reaching its maximum within 0.5 min (**Figures 6I, J**, **6i, j**).

*Average P_net_ over different time intervals.* In ‘Rex’, the average P_net_ during the first 10 min of the light period was highest in the ‘30 min/15 min’, ‘20 min/10 min’, and ‘10 min/5 min’ treatments, but was significantly reduced in the ‘4 h/2 h’ and ‘1 h/30 min’ treatments, with the lowest values observed in the ‘4 h/2 h’ treatment. (**Table 1**). This was consistent with the slower photosynthetic induction under treatments with less frequent light-dark cycles (and therefore longer dark period within each cycle) (**Figure 6**). As described in the ‘Materials and Methods’, this parameter was not measured in the treatments with long light-dark cycle durations (16 h/8 h’, ‘8 h/4 h’).

**Table 1:** Average leaf net photosynthetic rate (P_net_) during the first 10 min of the light period, the entire light period of one light-dark cycle, and the steady state rate for ‘Rex’ and ‘Rouxai’ under different light treatments.

Cultivar	P_net_ (µmol m^-2^ s^-1^)	16h/8h	8h/4h	4h/2h	1h/30min	30min/15min	20min/10min	10min/5min
‘Rex’	First 10-min average of the light period	—	—	5.5 ± 0.38 c	6.2 ± 0.28 bc	7.6 ± 0.19 a	7.5 ± 0.09 a	7.4 ± 0.32 ab
	Entire light period average for one light-dark cycle	—	—	—	8.6 ± 0.21 a	8.3 ± 0.24 a	8.0 ± 0.08 ab	7.4 ± 0.32 b
	Steady-state P_net_	10.0 ± 0.27 ab	10.1 ± 0.21 a	10.4 ± 0.16 a	9.5 ± 0.25 bc	9.3 ± 0.19 cd	8.6 ± 0.17 e	8.7 ± 0.32 de
‘Rouxai’	First 10-min average of the light period	—	—	2.8 ± 0.49 b	2.4 ± 0.43 b	4.7 ± 0.26 a	5.5 ± 0.29 a	5.7 ± 0.24 a
	Entire light period average for one light-dark cycle	—	—	—	5.7 ± 0.36 ^ns^	6.1 ± 0.25 ^ns^	6.2 ± 0.26 ^ns^	5.7 ± 0.24 ^ns^
	Steady-state P_net_	6.6 ± 0.28^ns^	6.8 ± 0.26^ns^	6.8 ± 0.17 ^ns^	6.9 ± 0.3 ^ns^	6.8 ± 0.27 ^ns^	6.9 ± 0.19 ^ns^	6.9 ± 0.17 ^ns^

Data represent mean ± SE (n=9; 3 plants per replicate x 3 replicate studies). Within each photosynthetic parameter, different letters indicate significant differences among treatments at *P* < 0.05. ^ns^ indicates non-significance at *P* < 0.05. For the16 h/8 h and 8 h/4 h treatments, only steady state P_net_ values were obtained due to the long light/dark cycle durations. See **Figure 1** legend for treatment details.

Similarly, the average P_net_ of ‘Rex’ over the entire light period was calculated only for the treatments with more frequent cycles (‘1 h/30 min’, ‘30min/15 min’, ‘20min/10 min’, and ‘10 min/5 min’). The ‘1 h/30 min’ treatment had the highest average P_net_ over the entire light period, although it did not differ statistically from the ‘30 min/15 min’ and ‘20 min/10 min’ treatment. The ‘10 min/5 min’ treatment showed the lowest average P_net_ over the entire light period, despite exhibiting rapid photosynthetic induction (**Table 1**; **Figure 6**).

Steady-state maximum P_net_ of ‘Rex’ was measured for all treatments and was highest in the ‘16 h/8 h’, ‘8 h/4 h’, and ‘4 h/2 h’ treatments. The ‘1 h/30 min’ treatment showed comparable steady-state values to the control treatment but was significantly lower than those in the ‘8 h/4 h’ and ‘4 h/2h’ treatments (**Table 1**). Steady-state P_net_ was further reduced in the ‘30 min/15 min’, ‘20 min/10 min’, and ‘10 min/5 min’ treatments, by 7%, 14%, and 13% relative to the control, respectively (**Table 1**).

In ‘Rouxai’, the average P_net_ during the first 10 min of the light period followed a similar response pattern to that of ‘Rex’, with the highest values in the ‘30 min/15 min’, ‘20 min/10 min’, and ‘10 min/5 min’ treatments and significantly lower values in the ‘4 h/2 h’ and ‘1 h/30 min’ treatments (**Table 1**). However, the average P_net_ over the entire light period and the steady-state maximum P_net_ showed no significant differences among treatments in ‘Rouxai’ (**Table 1**).

#### 3.3.2 Light response curves

**Figure 7:**
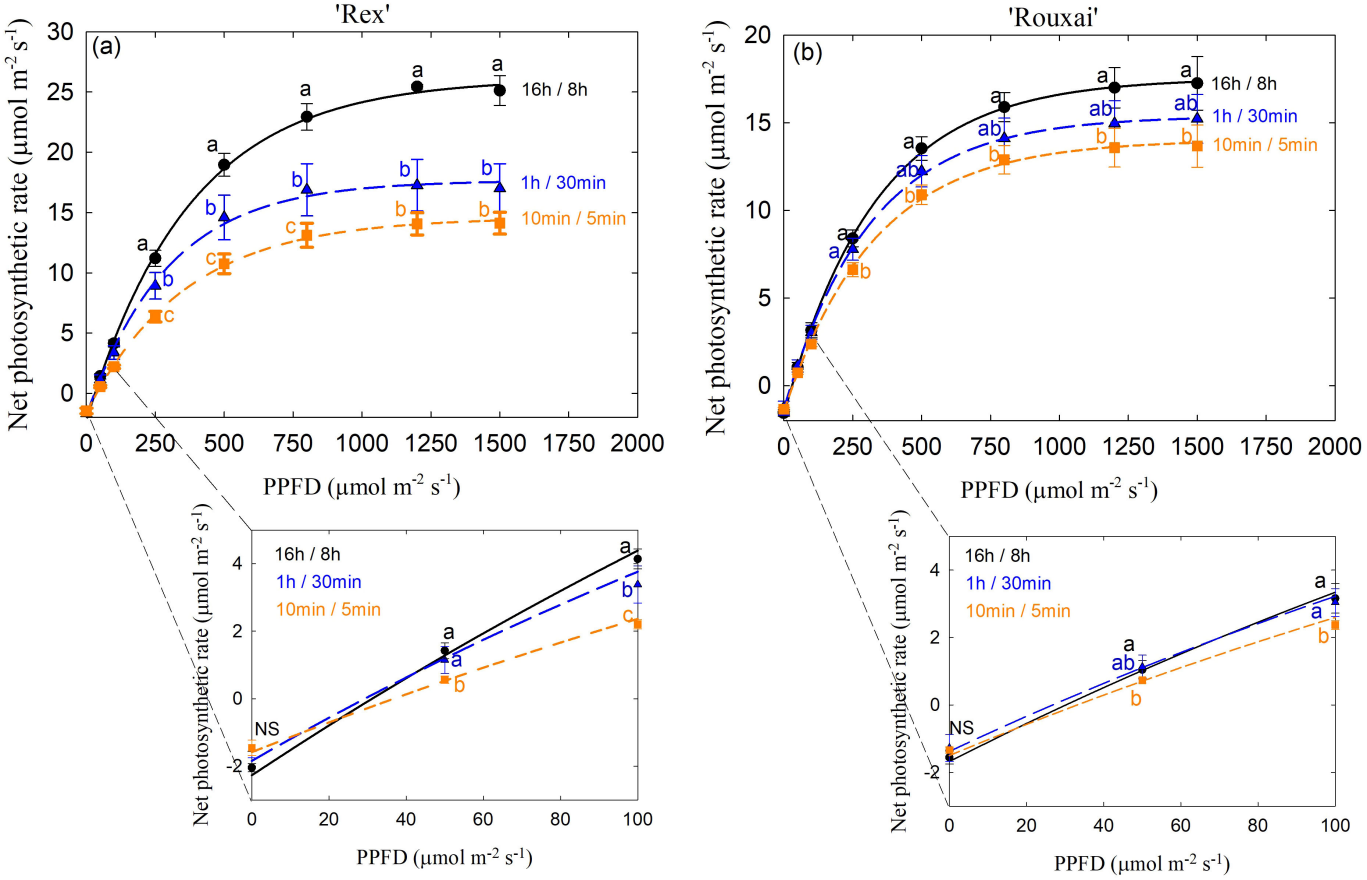
Leaf photosynthetic light response curves of ‘Rex’ **(a)** and ‘Rouxai’ **(b)** grown under the 16h/8h, 1h/30min, and 10min/5min light-dark cycle treatments. See **Figure 1** legend for treatment details. PPFD stands for photosynthetic photon flux density. Within each light level, different letters indicate significance at *P* < 0.05 among treatments with error bars representing SE (n=9).

Leaf light response curves were constructed for three treatments, ‘16 h/8 h’, ‘1 h/30 min’ and ‘10 min/5 min’, representing the least frequent, intermediate, and most frequent light-dark cycles, respectively (see Materials and Methods). In ‘Rex’, P_net_ was significantly reduced in the ‘1 h/30 min’ and ‘10 min/5 min’ treatments at all measured PPFD levels above 50 µmol m^-2^ s^-1^ compared to ‘16 h/8 h’ control, with the lowest P_net_ in the ‘10 min/5 min’ treatment at light levels above the light compensation point (**Figure 7a**). Similarly, P_net_ in the ‘10 min/5 min’ treatment was significantly reduced at all PPFD levels above 50 µmol m^-2^ s^-1^ compared to ‘16 h/8 h’ in ‘Rouxai’. However, there were no significant differences between the 16 h/8 h’ and ‘1 h/30 min’ treatments across all PPFD levels (**Figure 7b**).

#### 3.3.3 Maximum quantum yield of PSII

F_v_/F_m_ ranged from 0.807 to 0.819 in young plants and from 0.81 to 0.83 in mature plants across both cultivars, indicating an absence of photoinhibition in all treatments (**Supplementary Figure 4**).

## 4 Discussions

### 4.1 Growth reductions were mitigated under the most frequent light-dark cycles with short dark periods

Our results showed that total leaf area and shoot biomass were generally reduced when the frequency of light-dark cycles increased from 1 cycle (‘16 h/8 h’) to 48 cycles (‘20 min/10 min’) per 24-h in both ‘Rex’ and ‘Rouxai’. Notably, the most frequent light-dark cycles (96 cycles, ‘10 min/5 min’) resulted in growth comparable to the control in both cultivars (**Figures 4a-d**; **Supplementary Figure 2**). In general, frequent light-dark cycles tend to negatively affect plant growth, as plants are naturally adapted to a single light-dark cycle per 24 hours. Manipulation of light-dark cycles might alter various metabolic, morphological, and physiological processes and impair plant normal growth and development (Deepika et al., 2020; Guo et al., 2025; Hang et al., 2019; Zhou et al., 2022). For instance, Bhuiyan and van Iersel (2021) found that frequently alternating light between 400 µmol m^-2^ s^-1^ and darkness every 15 minutes resulted in significantly lower shoot biomass, total leaf area, and chlorophyll content in lettuce than constant light at 200 µmol m^-2^ s^-1^ over a 16-h photoperiod. Similarly, Kaiser et al. (2020) showed that fluctuating light intensities, applied by alternating light intensities between 900 µmol m^-2^ s^-1^ for 1 minute and 90 µmol m^-2^ s^-1^ for 4 minutes, significantly reduced the projected leaf area of *Arabidopsis* compared with plants grown under constant light providing the same daily light integral.

However, previous studies have found that plant growth is not always reduced under fluctuating light intensities or frequent light-dark cycles. Bonde (1955) grew plants under various light-dark cycles and found that 3 cycles per 24-h (4 h light/4 h dark) and 12 cycles per 24-h (1 h light/1 h dark) resulted in the greatest dry biomass in tomato seedlings and cocklebur plants, respectively. In both species, however, treatments with less frequent or more frequent cycles than these optima generally reduced growth compared with a single daily light-dark cycle (12h light/12h dark) (Bonde, 1955). Similarly, Chen and Yang (2018) reported that 4, 6 and 8 light-dark cycles significantly increased lettuce shoot and root fresh mass compared with one light-dark cycle per 24-h. Nevertheless, the underlying physiological mechanisms, such as photosynthetic responses, were not examined in these studies.

A recent study found that plant dry biomass and total leaf area of young tomato plants decreased linearly as the number of light-dark cycles increased from 1 to 6 cycles per 24-h (Yuan et al., 2025). This is largely consistent with the reduced lettuce growth observed in our study under treatments with up to 48 light-dark cycles. Slow photosynthetic induction (e.g., in the 4 h/2 h and 1 h/30 min treatments; **Figures 6A–D**) and/or reduced steady-state P_net_ (**Table 1**) most likely contributed to the observed growth reductions in these treatments. However, this reduction trend was mitigated in the treatment with the most frequent light-dark cycles (96 per 24-h; 10 min light/5 min dark) (**Figures 4a-d**; **Supplementary Figure 2**). This mitigation was likely due, at least in part, to rapid photosynthetic induction under this treatment (**Figure 6**), as discussed in detail in Section 4.2 below.

### 4.2 Duration of dark periods strongly affects photosynthetic induction kinetics and morphological acclimation in a cultivar-specific manner

Fluctuating light is generally thought to reduce overall carbon gain because of the lagged responses of stomata and the photosynthetic machinery to changes in light intensity (Kaiser et al., 2016; De Souza et al., 2020). However, the effects of fluctuating light on carbon assimilation depend strongly on the duration of the dark period within light-dark cycles, likely reflecting the combined effects of short-term photosynthetic induction kinetics and long-term acclimation adjustments. In our study, P_net_ increased rapidly following short dark periods in the 10 min light/5 min dark treatment and quickly reached steady state in both lettuce cultivars (**Figures 6I, J**). Similar rapid initial increases in P_net_ were observed in the ‘30 min/15 min’ and ‘20 min/10 min’ treatments during transitions from dark to light (**Figures 6E–H**).

Woodrow and Mott (1989) demonstrated that the exponential phase of photosynthetic induction, i.e., the initial period following a transition from darkness or low light to high light during which P_net_ increases rapidly and approximately exponentially, reflects the kinetics of Rubisco activation. After a step increase in light intensity following varying durations of darkness, the relaxation time of this exponential phase closely matched that of Rubisco activation. In addition, Rubisco deactivation in darkness was found to have a relaxation time of approximately 28 minutes (Woodrow and Mott, 1989). Thus, longer dark periods lead to greater Rubisco deactivation and consequently require more time for reactivation during dark to light transition. Consistent with this, slow photosynthetic induction over extended periods was observed in the ‘4 h/2 h’ and ‘1 h/30 min’ treatments (**Figures 6A–D**) in our study. In contrast, short durations (5 min or less) of dark period or low light intensity allow partial retention of Rubisco activation and have minimal impact on stomatal conductance (Lawson et al., 2010). Rubisco activation has been reported to be the major limitation to photosynthetic induction during the initial ~10 min of light in various species, including tobacco, rice and wheat (Tinoco-Ojanguren and Pearcy, 1993; Mott et al., 1997; Yamori et al., 2012; Taylor and Long, 2017). The short dark duration in the ‘10 min light/5 min dark’ treatment likely allowed partial activation of Rubisco and contributed to the rapid photosynthetic induction (**Figure 6**) and mitigated growth reductions (**Figure 4**; **Supplementary Figure 2**) observed in both lettuce cultivars. Additionally, stomatal conductance (g_s_) during the induction period, measured as the average g_s_ over the first 10 minutes of the light period, was significantly higher in the ‘10 min/5 min’ treatment than in the ‘4 h/2 h’ treatment (**Supplementary Table 1**), which may also have contributed to the fast photosynthetic induction after short dark periods.

In the ‘30 min/15 min’ and ‘20 min/10 min’ treatments, rapid initial increases in P_net_ were observed in both cultivars during light induction (**Figures 6E–H**). Similar to responses in the ‘10 min/5 min’ treatment, this rapid response likely results from partial retention of Rubisco activation, as complete Rubisco deactivation requires a longer dark period (~28 min; Woodrow and Mott, 1989). Notably, despite these rapid or comparable photosynthetic induction rates, both steady-state P_net_ and the average P_net_ of ‘Rex’ over the entire light period of a light-dark cycle generally decreased with increasing cycle frequency, reaching the lowest values in the ‘10 min/5min’ treatment (**Table 1**). Similarly, leaf photosynthetic capacity (expressed as maximum P_net_ at light-saturated intensity) in the ‘10 min/5min’ treatment was also significantly reduced (**Figure 7a**). Photoinhibition and low light use efficiency have been observed under fluctuating light in previous studies (Eberhard et al., 2008; Kaiser et al., 2016; Yamori et al., 2020). Although photoinhibition was not observed in our study (**Supplementary Figure 4**), it was possible that a higher fraction of absorbed light was dissipated via photoprotective mechanisms under frequent light-dark cycles, reducing leaf-level P_net_.

The reduced leaf P_net_ in ‘Rex’ under frequent light-dark cycles might also be associated with plant morphological acclimation (**Figures 4a, c**). Biochemical and morphological adjustments, including reduced leaf thickness and increased leaf area, have been observed in plants acclimated to light flecks or fluctuating light, which may lead to increased whole-plant light interception and partially compensate for reductions in leaf-level photosynthesis (Kaiser et al., 2016; Zhang et al., 2020). Similarly, we observed that ‘Rex’ showed reduced LMA but maintained a similar total leaf area in the ‘10 min/5 min’ treatment compared with the control (**Figures 4a, e**). In addition, biomass accumulation is often more strongly correlated with radiation capture at the whole-plant level and cumulative carbon gain rather than instantaneous leaf photosynthetic rate (Gifford et al., 1984). Rapid photosynthetic induction can improve cumulative CO_2_ assimilation during light fluctuations (Taylor and Long, 2017). Collectively, the comparable growth of ‘Rex’ in the ‘10 min/5 min’ treatment relative to the control could likely be attributed to the maintenance of total leaf area and rapid photosynthetic induction.

Although rapid initial photosynthetic inductions were observed in both cultivars in the ‘30 min/15 min’ and ‘20 min/10 min’ treatments, P_net_ in ‘Rex’ reached steady-state relatively quickly (within ~1 min), whereas in ‘Rouxai’ it continued to increase gradually over an additional ~20 minutes before reaching steady state (**Figures 6E–H**). This longer lagging time in ‘Rouxai’, a red-leafed cultivar, may be explained by a slower increase in effective light irradiation upon light induction due to light screening of anthocyanins (Gould, 2004), which could in turn delay biochemical processes such as Rubisco activation. However, stomatal limitations likely did not play a role, as stomatal conductance during the induction period in the ‘30 min/15 min’ and ‘20 min/10 min’ treatments was comparable to that in the ‘10 min/5 min’ treatment (**Supplementary Table 1**). Woodrow and Mott (1989) showed that as dark duration increased, the exponential phase of photosynthetic induction accounted for a greater proportion of the overall photosynthetic response in spinach, with a relaxation time of approximately 24 minutes. This is consistent with our observation that photosynthesis in ‘Rouxai’ continued to increase for ~20 minutes before reaching steady state in the ‘30 min/15 min’ and ‘20 min/10 min’ treatments (**Figures 6F, H**).

Despite the slower photosynthetic induction kinetics in ‘Rouxai’ under treatments with longer dark durations, steady-state P_net_ and average leaf P_net_ over the entire light period of a light/dark cycle were not negatively affected (**Table 1**). In contrast to ‘Rex’, which showed reduced LMA while maintaining large total leaf areas particularly at the mature plant stage (**Figures 4A, E**), ‘Rouxai’ exhibited increased leaf thickness, as indicated by higher LMA, while generally having significantly reduced total leaf area under more frequent light-dark cycles compared to the control (**Figures 4B, F**). Photosynthetic capacity is generally positively linked to LMA (Niinemets, 1999). The increased LMA in ‘Rouxai’ under more frequent light-dark cycles may have contributed to the minimal reduction in photosynthetic capacity in the ‘1 h/30 min’ treatment and the relatively small reduction observed in the ‘10 min/5 min’ treatment (**Figure 7b**). In contrast, ‘Rex’ showed larger reductions in photosynthetic capacity under these treatments relative to its control (**Figure 7a**). However, despite the higher LMA, shoot biomass of ‘Rouxai’ was generally reduced under treatments – compared with the control (**Figure 4D**). This was probably because the reduced leaf area offset the benefits of increased LMA at the whole-plant scale (**Figure 4b**). The comparable growth of ‘Rouxai’ in the 10 min/5 min treatment was most likely due to comparable integrated carbon assimilation driven by rapid photosynthetic induction combined with a similar total leaf area as the control.

### 4.3 Long-term acclimation effects on photosynthetic induction

Beyond the short-term effects of dark period duration on Rubisco activation and stomatal opening, plant long-term acclimation to fluctuating light might have also contributed to the varying photosynthetic induction rates in our treatments. Under long-term exposure to fluctuating light, plants develop systematic acclimation adjustments across different scales, from gene expression to leaf and whole-plant traits (Vialet-Chabrand et al., 2017; Schneider et al., 2019; Kaiser et al., 2016; Wei et al., 2021). In our study, increasing the frequency of light-dark cycles was associated with reduced concentrations of chlorophyll, carotenoids, and anthocyanins in both lettuce cultivars (**Figure 5**). Reduced chlorophyll content may lower light-harvesting capacity and reduce the initial excitation pressure on photosystems following dark-to-light transitions, potentially increasing the efficiency of electron transport and carbon assimilation during light induction (Maxwell et al., 1995). In addition, decreases in carotenoid and anthocyanin levels may indicate reduced photoprotective capacity, which could allow greater carbon assimilation during light induction under non-stressful conditions (Croce et al., 2024). Frequent light-dark cycles also caused changes in LMA, representing a major modification in leaf structure in both ‘Rex’ and ‘Rouxai’ (**Figures 4e, f**). Variations in LMA suggest structural acclimation that may alter leaf thickness, mesophyll organization, and internal CO_2_ diffusion pathways, thereby influencing diffusional limitations during light transitions (Evans et al., 2009; Tomas et al., 2013). Although beyond the scope of the study, the decreases in LMA in ‘Rex’ under frequent light-dark cycles could be partially associated with improved mesophyll conductance, photosynthetic electron transport and Rubisco carboxylation activity (Niinemets et al., 2005). In contrast, the increases in LMA in ‘Rouxai’ under frequent light-dark cycles may have contributed to its relatively slower photosynthetic induction compared with ‘Rex’. Collectively, these biochemical and structural differences may help explain the slower induction rates under treatments with less frequent light-dark cycles, as well as cultivar-specific differences in induction kinetics.

### 4.4 Frequent light-dark cycles reduced pigment concentrations in lettuce

Frequent light-dark cycles resulted in significant reductions in pigment concentrations in both lettuce cultivars. Concentrations of chlorophylls (Chlorophyll a, b, and total chlorophylls) and total carotenoids were significantly lower under the ‘30 min/15 min’, ‘20 min/10 min’, and ‘10 min/5 min’ treatments in both cultivars compared with the control (**Figures 5a-d**; **Supplementary Figures 3a–d**). Chlorophylls are the primary photosynthetic pigments, whereas carotenoids act as accessory pigments for light harvesting and photoprotection. Therefore, carotenoids biosynthesis is typically tightly coordinated with chlorophyll accumulation (Siefermann-Harms, 1987; Goodwin and Britton, 1988; Kong and Nemali, 2021). Pigment accumulation in plants is determined by the rates of synthesis and degradation, which are regulated by both internal circadian rhythms and external environmental cues, including light-dark cycles (Stanley and Yuan, 2019; García-Caparr’os et al., 2020). Many key genes involved in chlorophylls and carotenoids biosynthesis are strongly controlled by circadian rhythms (Kloppstech, 1985; Papenbrock et al., 1999; Khan et al., 2010; Hu et al., 2024), and changes in light-dark cycles reset the internal rhythms (Wang et al., 2022). The frequent, short duration light-dark cycles applied in our study might have disrupted the coordination between internal circadian regulation and external cues, therefore disturbing the balance between synthesis and degradation of chlorophylls and carotenoids. In addition, the biosynthesis of chlorophylls requires sustained illumination (Bennett, 1981). Previous studies have found that frequent light-dark cycles can damage the Chl a/b protein complex in maize and tomato plants (Maróti, 1982) and impair the photosynthetic apparatus in beans (Takacs and Maroti, 1984). Consistently, reductions in chlorophyll and carotenoid concentration under frequent light-dark cycles or fluctuating light intensities have been reported in other species (Zhang et al., 2020; Wei et al., 2021; Yuan et al., 2025).

Importantly, the observed decline in chlorophyll and carotenoid concentration was unlikely to result from photobleaching or photodamage. Although photodamage has been observed under extreme fluctuating light conditions where light intensity changes tremendously within seconds or minutes (Kono et al., 2017; Shi et al., 2022), the light-dark cycle treatments used in our study (‘30 min/15 min’, ‘20 min/10 min’, and ‘10 min/5 min’) contained relatively low light intensity and did not impose extreme fluctuations. Moreover, high F_v_/F_m_ values (above 0.807 in all treatments) indicated an absence of photosystem II photoinhibition in our study (**Supplementary Figure 4**), further supporting that the reductions in chlorophylls and carotenoids were not due to damages to the photosynthetic apparatus.

Similarly, anthocyanins concentrations were significantly reduced under the ‘30 min/15 min’, ‘20 min/10 min’, and ‘10 min/5 min’ treatments in ‘Rouxai’ (**Figures 5e, f**; **Supplementary Figure 3e**, f). Anthocyanins function as ‘sunscreen’ pigments that protect plants against photoinhibition and photooxidative damage under high light stress (Gould, 2004). Therefore, increases in anthocyanin concentration are usually associated with high-light exposure or other stress conditions (such as cold temperature) that reduce light use efficiency (Zeng et al., 2010; Shao et al., 2007). However, neither photoinhibition nor stress-induced reductions in photosynthetic activity were observed in ‘Rouxai’ as indicated by high F_v_/F_m_ values (**Supplementary Figure 4**) and steady-state P_net_ (**Table 1**). Instead, the decreases in anthocyanin concentrations under frequent light-dark cycles were most likely a result of repeated interruptions of the light and dark periods, as previous studies have found that sustained light exposure is required for anthocyanins production (Downs and Siegelman, 1963; Downs, 1964; Lu et al., 2015). Reduced anthocyanin accumulation may also have contributed to the maintenance of high leaf-level photosynthesis under frequent light-dark cycles (**Table 1**). Because red coloration is often perceived as a quality attribute in red lettuce cultivars, the effects of light-dark cycles on anthocyanin concentration should be considered when implementing dynamic lighting strategies for indoor lettuce production.

## 5 Conclusions

Our study showed that frequent light-dark cycles generally reduced growth and pigment concentrations in lettuce, even when the same daily light integral was provided across treatments. However, growth reductions were mitigated under the most frequent light-dark cycles with short dark durations (5 min) in both cultivars, mainly due to the rapid photosynthetic induction following short dark periods and the maintenance of a high total leaf area. Cultivar-specific photosynthetic and morphological acclimation strategies were observed in response to frequent light-dark cycles. Because the duration of darkness or low light can strongly affect photosynthetic induction kinetics, steady-state photosynthetic rates, key morphological traits such as total leaf area, leaf thickness and pigment concentration, and ultimately biomass accumulation, it should be carefully considered when implementing fluctuating light regimes in controlled environment crop production. Further research is needed to identify dark-duration threshold that minimally impact photosynthesis and growth for different crops in controlled environments.

## Data Availability

The original contributions presented in the study are included in the article/**Supplementary Material**. Further inquiries can be directed to the corresponding author.

## Supplementary material

Supplementary Table 1 Average stomatal conductance (gs) under different light-dark cycles.

Supplementary Figure 1 Image analysis of normalized difference anthocyanin index (NDAI).

Supplementary Figure 2 Shoot fresh mass of ‘Rex’ and ‘Rouxai’ under different light-dark cycles.

Supplementary Figure 3 Pigment concentrations of ‘Rex’ and ‘Rouxai’ at the mature plant stage.

Supplementary Figure 4 Maximum quantum efficiency (F_v_/F_m_) of ‘Rex’ and ‘Rouxai’ at young and mature plant stages.

## References

[B1] Bennett J. (1981). Biosynthesis of the light-harvesting chlorophyll a/b protein. Eur. J. Biochem. 118, 61–70. 7026240 10.1111/j.1432-1033.1981.tb05486.x

[B2] Bhuiyan R. van Iersel M. W. (2021). Only extreme fluctuations in light levels reduce lettuce growth under sole source lighting. Front. Plant Sci. 12. doi: 10.3389/fpls.2021.619973 33584773 PMC7875872

[B3] Bonde E. K. (1955). The effect of various cycles of light and darkness on the growth of tomato and cocklebur plants. Physiol. Plant 8, 913–923. doi: 10.1111/j.1399-3054.1955.tb07786.x 41834780

[B4] Chazdon R. L. Pearcy R. W. (1986). Photosynthetic responses to light variation in rain-forest species. 1. Induction under constant and fluctuating light conditions. Oecologia. 69, 517–523. doi: 10.1007/bf00410357 28311610

[B5] Chen X. L. Yang Q. C. (2018). Effects of intermittent light exposure with red and blue light emitting diodes on growth and carbohydrate accumulation of lettuce. Sci. Hortic. 234, 220–226. doi: 10.1016/j.scienta.2018.02.055 41847267

[B6] Croce R. Carmo-Silva E. Cho Y. B. Ermakova M. Harbinson J. Lawson T. . (2024). Perspectives on improving photosynthesis to increase crop yield. Plant Cell 36, 3944–3973. doi: 10.1093/plcell/koae132 38701340 PMC11449117

[B7] Deepika A. Sagar S. Singh A. (2020). Dark-induced hormonal regulation of plant growth and development. Front. Plant Sci. 11. doi: 10.3389/fpls.2020.581666 33117413 PMC7575791

[B8] De Souza A. P. Wang Y. Orr D. J. Carmo-Silva E. Long S. P. (2020). Photosynthesis across African cassava germplasm is limited by Rubisco and mesophyll conductance at steady state, but by stomatal conductance in fluctuating light. New Phytol. 225, 2498–2512. doi: 10.1111/nph.16142 31446639 PMC7065220

[B9] Downs R. J. (1964). Photocontrol of anthocyanin synthesis. J. Wash. Acad. Sci. 54, 112–120. doi: 10.1104/pp.38.1.25 16655747 PMC549873

[B10] Downs R. J. Siegelman H. W. (1963). Photocontrol of anthocyanin synthesis in milo seedlings. Plant Physiol. 38, 25–30. doi: 10.1104/pp.38.1.25 16655747 PMC549873

[B11] Eberhard S. Finazzi G. Wollman F. A. (2008). The dynamics of photosynthesis. Annu. Rev. Genet. 42, 463–515. doi: 10.1146/annurev.genet.42.110807.091452 18983262

[B12] Evans J. R. Kaldenhoff R. Genty B. Terashima I. (2009). Resistances along the CO_2_ diffusion pathway inside leaves. J. Exp. Bot. 60, 2235–2248. doi: 10.1093/jxb/erp117 19395390

[B13] García-Caparrós P. Sabio F. Barbero F. J. Chica R. M. Lao M. T. (2020). Physiological responses of tomato and cucumber seedlings under different light-dark cycles. Agronomy 10, 945.

[B14] Gifford R. M. Thorne J. H. Hitz W. D. Giaquinta R. T. (1984). Crop productivity and photoassimilate partitioning. Science. 225, 801–808. doi: 10.1126/science.225.4664.801 17801136

[B15] Goodwin T. Britton G. W. (1988). “ Distribution and analysis of carotenoids,” in Plant pigments. Ed. Goodwin T. W. ( Academic Press, London), 62–132.

[B16] Gould K. S. Markham K. R. Smith R. H. Goris J. J. (2000). Functional role of anthocyanins in the leaves of Quintinia serrata A. Cunn. J. Exp. Bot. 51, 1107–1115. 10948238 10.1093/jexbot/51.347.1107

[B17] Gould K. S. (2004). Nature's Swiss army knife: the diverse protective roles of anthocyanins in leaves. J. Biomed. Biotechnol. 5, 314–320. doi: 10.1016/b978-0-12-394807-6.00229-x 15577195 PMC1082902

[B18] Guo K. Guo Z. (2025). Effect of different light–dark cycles on the growth and nutritional quality of celery. Agriculture 15, 2228.

[B19] Hang T. Lu N. Takagaki M. Mao H. (2019). Leaf area model based on thermal effectiveness and photosynthetically active radiation in lettuce grown in mini-plant factories under different light cycles. Sci. Hortic. 252, 113–120. doi: 10.1016/j.scienta.2019.03.057 41847267

[B20] Hu Z. H. Sun M. Z. Yang K. X. Zhang N. Chen C. Xiong J. W. . (2024). High-throughput transcriptomic analysis of circadian rhythm of chlorophyll metabolism under different photoperiods in tea plants. Int. J. Mol. Sci. 25, 9270. doi: 10.3390/ijms25179270 39273222 PMC11395263

[B21] Kaiser E. Matsubara S. Harbinson J. Heuvelink E. Marcelis L. F. M. (2018). Acclimation of photosynthesis to lightflecks in tomato leaves: Interaction with progressive shading in a growing canopy. Physiol. Plant 162, 506–517. doi: 10.1111/ppl.12668 29125181

[B22] Kaiser E. Morales A. Harbinson J. Heuvelink E. Prinzenberg A. E. Marcelis L. F. (2016). Metabolic and diffusional limitations of photosynthesis in fluctuating irradiance in Arabidopsis thaliana. Sci. Rep. 6, 31252. doi: 10.1038/srep31252 27502328 PMC4977489

[B23] Kaiser E. Walther D. Armbruster U. (2020). Growth under fluctuating light reveals large trait variation in a panel of Arabidopsis accessions. Plants. 9, 316. doi: 10.3390/plants9030316 32138234 PMC7154909

[B24] Khan S. Rowe S. C. Harmon F. G. (2010). Coordination of the maize transcriptome by a conserved circadian clock. BMC Plant Biol. 10, 126. doi: 10.1186/1471-2229-10-126 20576144 PMC3095283

[B25] Kim C. van Iersel M. W. (2023). Image-based phenotyping to estimate anthocyanin concentrations in lettuce. Front. Plant Sci. 14, 1155722. 37077649 10.3389/fpls.2023.1155722 PMC10109386

[B26] Kloppstech K. (1985). Diurnal and circadian rhythmicity in the expression of light-induced plant nuclear messenger RNAs. Planta. 165, 502–506. doi: 10.1007/bf00398095 24241223

[B27] Kong Y. Nemali K. (2021). Blue and far-red light affect area and number of individual leaves to influence vegetative growth and pigment synthesis in lettuce. Front. Plant Sci. 12. doi: 10.3389/fpls.2021.667407 34305967 PMC8297648

[B28] Kono M. Terashima I. (2014). Long-term and short-term responses of the photosynthetic electron transport to fluctuating light. J. Photochem. Photobiol. B 137, 89–99. doi: 10.1016/j.jphotobiol.2014.02.016 24776379

[B29] Kono M. Yamori W. Suzuki Y. Terashima I. (2017). Photoprotection of PSI by far-red light against the fluctuating light-induced photoinhibition in Arabidopsis thaliana and field-grown plants. Plant Cell Physiol. 58, 35–45. doi: 10.1093/pcp/pcw215 28119424

[B30] Kozai T. (2022). “ Contribution of PFALs to the sustainable development goals and beyond,” in Plant factory basics, applications and advances. Eds. Kozai T. Niu G. Masabni J. G. ( Academic Press, London), 57–79. doi: 10.1016/B978-0-323-85152-7.00016-1, PMID:

[B31] Kromdijk J. Głowacka K. Leonelli L. Gabilly S. T. Iwai M. Niyogi K. K. . (2016). Improving photosynthesis and crop productivity by accelerating recovery from photoprotection. Science. 354, 857–861. doi: 10.1126/science.aai8878 27856901

[B32] Lawson T. von Caemmerer S. Baroli I. (2010). Photosynthesis and stomatal behaviour. Prog. Bot. 72, 265–304.

[B33] Long S. P. Taylor S. H. Burgess S. J. Carmo-Silva E. Lawson T. De Souza A. P. . (2022). Into the shadows and back into sunlight: Photosynthesis in fluctuating light. Annu. Rev. Plant Biol. 73, 617–648. doi: 10.1146/annurev-arplant-070221-024745 35595290

[B34] Lu Y. Zhang M. Meng X. Wan H. Zhang J. Tian J. . (2015). Photoperiod and shading regulate coloration and anthocyanin accumulation in the leaves of malus crabapples. Plant Cell. Tiss Org. 121, 619–632. doi: 10.1007/s11240-015-0733-3 41841152

[B35] Maróti I. (1982). Effect of short light-dark cycles on the chlorophyll and carotenoid content of maize and tomatoes. Acta Biol. Szeged. 2, 85–94.

[B36] Maxwell D. P. Falk S. Huner N. P. (1995). Photosystem II excitation pressure and development of resistance to photoinhibition I. Light-harvesting complex II abundance and zeaxanthin content in Chlorella vulgaris. Plant Physiol. 107, 687–694. doi: 10.1104/pp.107.3.687 12228392 PMC157183

[B37] Mott K. A. Snyder G. W. Woodrow I. E. (1997). Kinetics of Rubisco activation as determined from gas-exchange measurements in antisense plants of Arabidopsis thaliana containing reduced levels of Rubisco activase. Aust. J. Plant Physiol. 24, 811–818. doi: 10.1071/pp97071 41161682

[B38] Niinemets Ü. (1999). Research review. Components of leaf dry mass per area–thickness and density–alter leaf photosynthetic capacity in reverse directions in woody plants. New Phytol. 144, 35–47. doi: 10.1046/j.1469-8137.1999.00466.x 41717205

[B39] Niinemets Ü. Cescatti A. Rodeghiero M. Tosens T. (2005). Leaf internal diffusion conductance limits photosynthesis more strongly in older leaves of Mediterranean evergreen broad-leaved species. Plant Cell Environ. 28, 1552–1566. doi: 10.1111/j.1365-3040.2005.01392.x 41834780

[B40] Papenbrock J. Mock H. P. Kruse E. Grimm B. (1999). Expression studies in tetrapyrrole biosynthesis: Inverse maxima of magnesium chelatase and ferro chelatase activity during cyclic photoperiods. Planta. 208, 264–273. doi: 10.1007/s004250050558 41841152

[B41] Pearcy R. W. (1990). Sunflecks and photosynthesis in plant canopies. Annu. Rev. Plant Biol. 41, 421–453. doi: 10.1146/annurev.arplant.41.1.421 41139587

[B42] Pearcy R. W. Krall J. P. Sassenrath-Cole G. F. (1996). “ Photosynthesis in fluctuating light environments,” in Photosynthesis and the Environment. Ed. Baker N. R. ( Kluwer Academic, Dordrecht), 321–346.

[B43] Roach T. Krieger-Liszkay A. (2014). Regulation of photosynthetic electron transport and photoinhibition. Curr. Protein Pept. Science. 15, 351–362. doi: 10.2174/1389203715666140327105143 24678670 PMC4030316

[B44] Schneider T. Bolger A. Zeier J. Preiskowski S. Benes V. Trenkamp S. . (2019). Fluctuating light interacts with time of day and leaf development stage to reprogram gene expression. Plant Physiol. 179, 1632–1657. doi: 10.1104/pp.18.01443 30718349 PMC6446761

[B45] Shao L. Shu Z. Sun S. L. Peng C. L. Wang X. J. Lin Z. F. . (2007). Antioxidation of anthocyanins in photosynthesis under high temperature stress. J. Integr. Plant Biol. 49, 1341–1351. doi: 10.1016/j.febslet.2014.09.018 25261252

[B46] Shi Q. Wang X. Zeng Z. Huang W. (2022). Photoinhibition of photosystem I induced by different intensities of fluctuating light is determined by the kinetics of ΔpH formation rather than linear electron flow. Antioxidants. 11, 2325. doi: 10.3390/antiox11122325 36552532 PMC9774317

[B47] Siefermann-Harms D. (1987). The light-harvesting and protective functions of carotenoids in photosynthetic membranes. Physiol. Plant 69, 561–568. doi: 10.1111/j.1399-3054.1987.tb09240.x 41834780

[B48] Stanley L. Yuan Y. W. (2019). Transcriptional regulation of carotenoid biosynthesis in plants: So many regulators, so little consensus. Front. Plant Sci. 10. doi: 10.3389/fpls.2019.01017 31447877 PMC6695471

[B49] Takács E. Maróti I. (1984). The effect of short light-dark cycles on the membrane system of bean chloroplasts. Acta Biol. Szeged. 30, 61–73.

[B50] Taylor S. Long S. P. (2017). Slow induction of photosynthesis on shade to sun transitions in wheat may cost at least 21% of productivity. Philos. Trans. R. Soc. B. Biol. Sci. 372, 20160543. doi: 10.1098/rstb.2016.0543 28808109 PMC5566890

[B51] Tinoco-Ojanguren C. Pearcy R. W. (1993). Stomatal dynamics and its importance to carbon gain in two rainforest Piper species. Oecologia. 94, 395–402. doi: 10.1007/BF00317115 28313677

[B52] Tomas M. Flexas J. Copolovici L. Galmes J. Hallik L. Medrano H. . (2013). Importance of leaf anatomy in determining mesophyll diffusion conductance to CO_2_ across species: quantitative limitations and scaling up by models. J. Exp. Bot. 64, 2269–2281. doi: 10.1093/jxb/ert086, PMID: 23564954 PMC3654418

[B53] Vialet-Chabrand S. Matthews J. S. Simkin A. J. Raines C. A. Lawson T. (2017). Importance of fluctuations in light on plant photosynthetic acclimation. Plant Physiol. 173, 2163–2179. doi: 10.1104/pp.16.01767 28184008 PMC5373038

[B54] Wang S. Steed G. Webb A. A. (2022). Circadian entrainment in arabidopsis. Plant Physiol. 190, 981–993. doi: 10.1093/plphys/kiac204 35512209 PMC9516740

[B55] Ware M. A. Belgio E. Ruban A. V. (2015). Photoprotective capacity of non-photochemical quenching in plants acclimated to different light intensities. Photosynth. Res. 126, 261–274. doi: 10.1007/s11120-015-0102-4 25702085

[B56] Wei Z. Duan F. Sun X. Song X. Zhou W. (2021). Leaf photosynthetic and anatomical insights into mechanisms of acclimation in rice in response to long-term fluctuating light. Plant Cell Environ. 44, 747–761. doi: 10.1111/pce.13954 33215722

[B57] Wellburn A. R. (1994). The spectral determination of chlorophylls a and b, as well as total carotenoids, using various solvents with spectrophotometers of different resolution. J. Plant Physiol. 144, 307–313.

[B58] Woodrow I. E. Mott K. A. (1989). Rate limitation of non-steady-state photosynthesis by ribulose-1, 5-bisphosphate carboxylase in spinach. Aust. J. Plant Physiol. 16, 487–500. doi: 10.1071/pp9890487 41161682

[B59] Yamori W. Kusumi K. Iba K. Terashima I. (2020). Increased stomatal conductance induces rapid changes to photosynthetic rate in response to naturally fluctuating light conditions in rice. Plant Cell Environ. 43, 1230–1240. doi: 10.1111/pce.13725 31990076

[B60] Yamori W. Masumoto C. Fukayama H. Makino A. (2012). Rubisco activase is a key regulator of non-steady-state photosynthesis at any leaf temperature and to a lesser extent, of steady-state photosynthesis at high temperature. Plant J. 71, 871–880. doi: 10.1111/j.1365-313x.2012.05041.x 22563799

[B61] Yuan X. Bian Z. Marcelis L. F. M. Yang Q. Heuvelink E. (2025). Six light/dark cycles within 24 h reduce tomato plant growth primarily resulting from a short uninterrupted dark period. Sc. Hortic. 344, 114047. doi: 10.1016/j.scienta.2025.114047 41847267

[B62] Zeng X. Q. Chow W. S. Su L. J. Peng X. X. Peng C. L. (2010). Protective effect of supplemental anthocyanins on Arabidopsis leaves under high light. Physiol. Plant 138, 215–225. doi: 10.1111/j.1399-3054.2009.01316.x 19947975

[B63] Zhang Y. Kaiser E. Marcelis L. F. M. Yang Q. Li T. (2020). Salt stress and fluctuating light have separate effects on photosynthetic acclimation, but interactively affect biomass. Plant Cell Environ. 43, 2192–2206. doi: 10.1111/pce.13810 32463133

[B64] Zhou J. Wang J. Hang T. Li P. (2022). Photosynthetic characteristics and growth performance of lettuce (Lactuca sativa L.) under different light/dark cycles in mini plant factories. Photosynthetica. 58, 740–747. doi: 10.32615/ps.2020.013

