# Supplementary material for: Rapid photosynthetic induction after short dark periods mitigates growth reduction in lettuce exposed to frequent light-dark cycles
Source: Front Plant Sci. 2026 Mar 30;17:1791561. doi: 10.3389/fpls.2026.1791561 (PMC13070824; doi:10.3389/fpls.2026.1791561)
Supplement: Supplementary Table 1 — Average stomatal conductance (gs) under different light-dark cycles. [file DataSheet1.pdf]

Supplementary Material

Rapid photosynthetic induction after short dark periods mitigates growth reduction in lettuce exposed to frequent light-dark cycles

Yuyao Kong[1,2], Shuyang Zhen[1]

[1]Department of Horticultural Sciences, Texas A&M University, College Station, TX, 77843, USA

[2]Department of Horticultural Sciences, University of Florida, Gainesville, FL, 32611, USA

Supplementary Table 1. Average stomatal conductance (g_s) during the first 10 min of the light period and the entire light period of one light-dark cycle for 'Rex' and 'Rouxai' under different light treatments. Data represent mean ± SE (n=9; 3 plants per replicate x 3 replicate studies). Within each row, different letters indicate significant differences among treatments at P < 0.05. ^{ns} indicates non-significance at P <0.05. Data for the the16 h / 8 h and 8 h / 4 h treatments were not measured due to the long light/dark cycle durations. For the 4 h / 2 h treatment, gs was only obtained for the first 10 min of the light period. See Figure 1 legend for treatment details.

Cultivar	g_s (mol m^{-2} s^{-1})	16h/8h	8h/4h	4h/2h	1h/30min	30min/15min	20min/10min	10min/5min
'Rex'	First 10-min average of the light period	—	—	0.05 ± 0.005 c	0.08 ± 0.011 b	0.12 ± 0.007 a	0.13 ± 0.007 a	0.11 ± 0.008 ab
	Entire light period average for one light-dark cycle	—	—	—	0.13 ± 0.01^{ns}	0.14 ± 0.007^{ns}	0.15 ± 0.009^{ns}	0.12 ± 0.008^{ns}
'Rouxai'	First 10-min average of the light period	—	—	0.04 ± 0.011 c	0.03 ± 0.006 bc	0.08 ± 0.011 ab	0.11 ± 0.011 a	0.12 ± 0.023 a
	Entire light period average for one light-dark cycle	—	—	—	0.12 ± 0.016^{ns}	0.13 ± 0.016^{ns}	0.16 ± 0.014^{ns}	0.12± 0.023^{ns}

1 Supplementary Figures

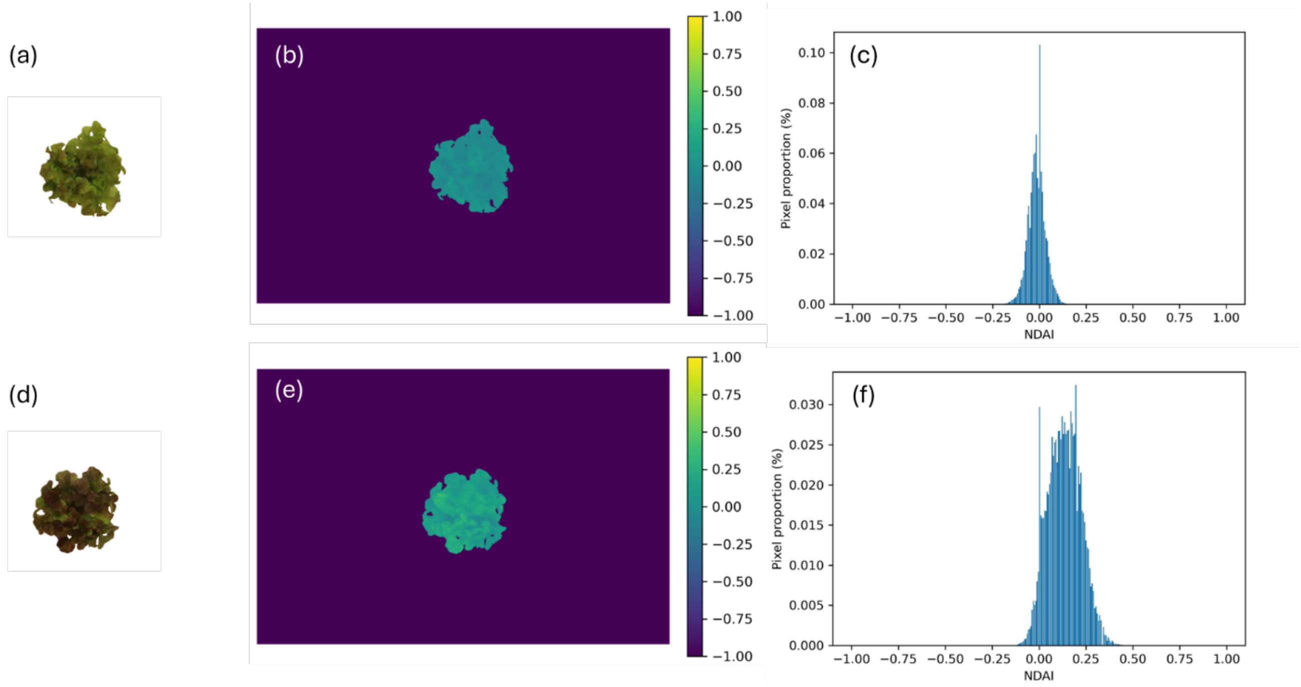

Supplementary Figure 1. Image analysis of normalized difference anthocyanin index (NDAI). Panels (a) and (d) show representative RGB images of lettuce 'Rouxai' with low and high anthocyanin content, respectively. (b) and (e) show the corresponding NDAI images. (c) and (f) show the corresponding pixel-value histograms.

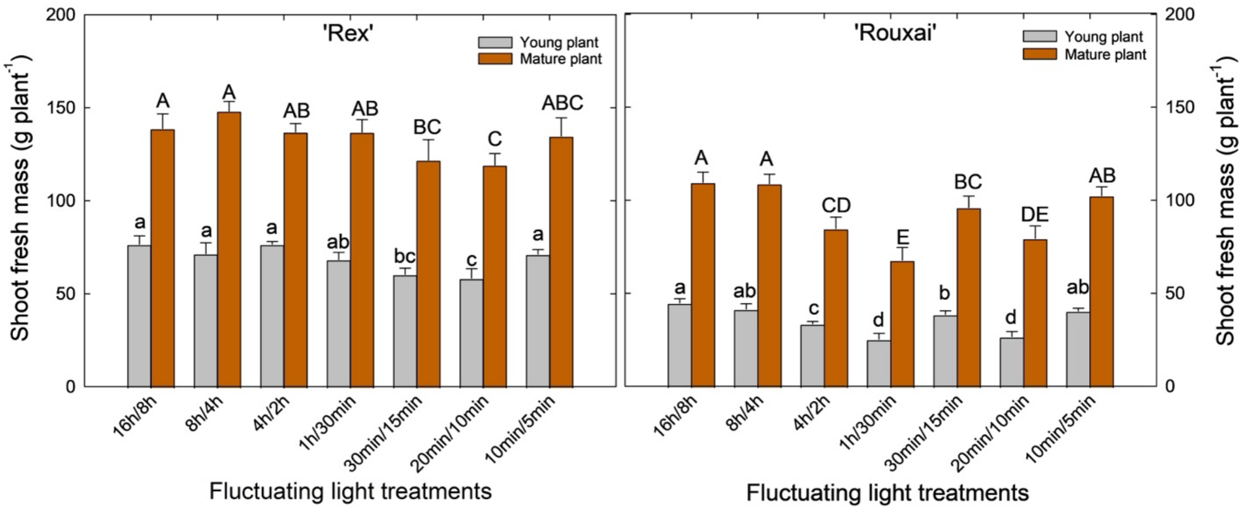

Supplementary Figure 2. Shoot fresh mass of 'Rex' and 'Rouxai' under different light-dark cycles. See Figure 1 legend for treatment details. Within each growth stage, different letters indicate significance at $P < 0.05$ among the treatments with error bars representing SE (n = 9; 3 plants per replicate x 3 replicate studies).

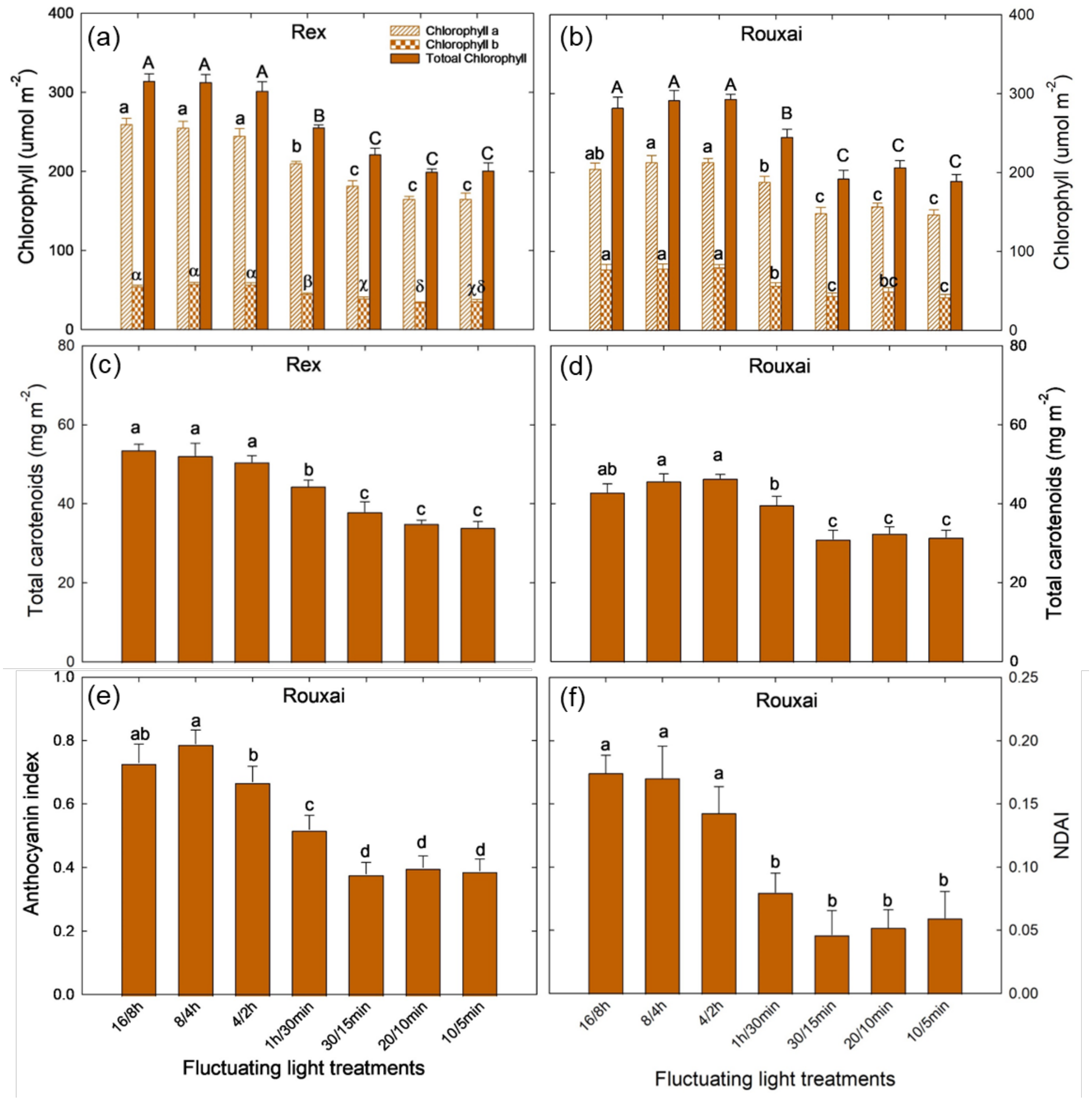

Supplementary Figure 3. Chlorophylls concentration (a-b) and total carotenoids concentration (c-d) of 'Rex' (a and c) and 'Rouxai' (b and d) at the mature plant stage, as well as extraction-based anthocyanin index (e) and image-based normalized difference anthocyanin index (NDAI) (f) of 'Rouxai' mature plants under different light-dark cycles. See Figure 1 legend for treatment details. Different letters indicate significance at $P < 0.05$ among the treatments with error bars representing SE (n = 9; 3 plants per replicate x 3 replicate studies).

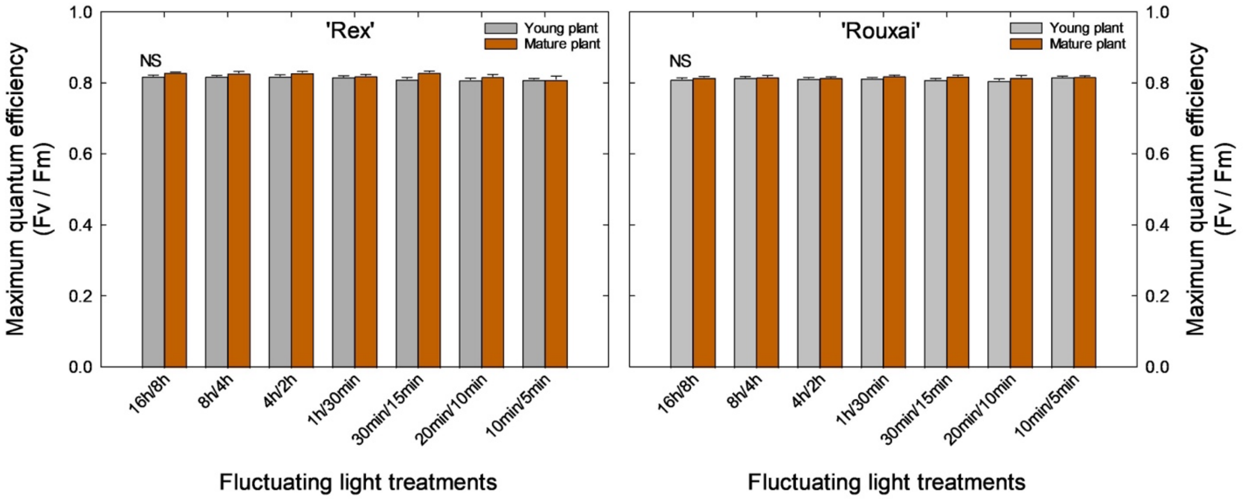

Supplementary Figure 4. Maximum quantum efficiency (F_v/F_m) of 'Rex' and 'Rouxai' at young and mature plant stages. Data represent mean ± SE (n=9; 3 plants per replicate x 3 replicate studies). NS indicates non-significance at *P* <0.05.
